# Quantitative assessment of computational models for retinotopic map formation

**DOI:** 10.1002/dneu.22241

**Published:** 2014-11-14

**Authors:** J J Johannes Hjorth, David C Sterratt, Catherine S Cutts, David J Willshaw, Stephen J Eglen

**Affiliations:** ^1^ Cambridge Computational Biology Institute, Department of Applied Mathematics and Theoretical PhysicsUniversity of CambridgeCambridgeCB3 0WAUnited Kingdom; ^2^ Institute for Adaptive and Neural Computation, School of InformaticsUniversity of EdinburghEdinburghEH8 9ABUnited Kingdom

**Keywords:** mouse, retinocollicular projection, retinotopic map formation, computational modelling framework, quantitative evaluation

## Abstract

Molecular and activity‐based cues acting together are thought to guide retinal axons to their terminal sites in vertebrate optic tectum or superior colliculus (SC) to form an ordered map of connections. The details of mechanisms involved, and the degree to which they might interact, are still not well understood. We have developed a framework within which existing computational models can be assessed in an unbiased and quantitative manner against a set of experimental data curated from the mouse retinocollicular system. Our framework facilitates comparison between models, testing new models against known phenotypes and simulating new phenotypes in existing models. We have used this framework to assess four representative models that combine Eph/ephrin gradients and/or activity‐based mechanisms and competition. Two of the models were updated from their original form to fit into our framework. The models were tested against five different phenotypes: wild type, *Isl2‐EphA3* ^ki/ki^, *Isl2‐EphA3* ^ki/+^, *ephrin‐A2,A3,A5* triple knock‐out (TKO), and *Math5* ^−/−^ (*Atoh7*). Two models successfully reproduced the extent of the *Math5* ^−/−^ anteromedial projection, but only one of those could account for the collapse point in *Isl2‐EphA3* ^ki/+^. The models needed a weak anteroposterior gradient in the SC to reproduce the residual order in the *ephrin‐A2,A3,A5* TKO phenotype, suggesting either an incomplete knock‐out or the presence of another guidance molecule. Our article demonstrates the importance of testing retinotopic models against as full a range of phenotypes as possible, and we have made available MATLAB software, we wrote to facilitate this process. © 2014 Wiley Periodicals, Inc. Develop Neurobiol 75: 641–666, 2015

## INTRODUCTION

**Figure 1:**
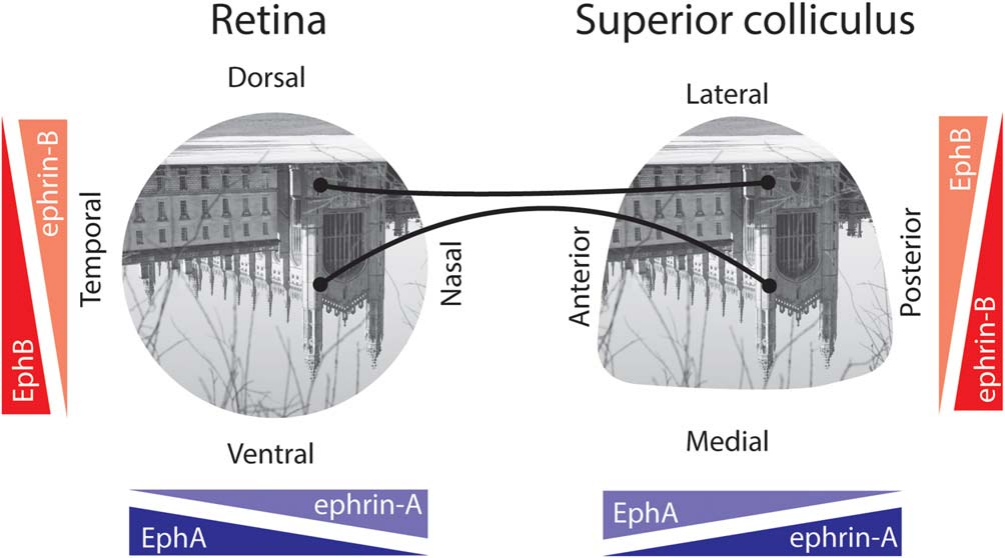
Schematic of retinotopic map formation. Retinal neurons project to SC in a topographic fashion. Each axis has an independent set of gradients instructing the map formation. Eph receptors of two different families are expressed across the retina in a graded fashion. The orthogonal A and B systems operate in distinct ways, with the gradients in the retina and SC matching up in opposite direction (A system high to low, B system high to high). The retinal EphA receptor gradient is low nasally and high temporally, whereas the retinal ephrin‐A ligand countergradient has the opposite direction. In the SC, the ephrin‐A ligand gradient goes from low anterior to high posterior, while the EphA receptor countergradient in the SC is in the opposite direction. The retinal EphB receptor gradient goes from ventral (high) to dorsal (low), while the ephrin‐B ligand countergradient is in the opposite direction (McLaughlin and O'Leary, 2005). In the SC, the ephrin‐B ligand gradient goes medial (high) to lateral (low), and the EphB receptor countergradient has the reverse slope (Hindges et al., 2002; McLaughlin and O'Leary, 2005).

Many sensory systems are organized into topographic maps, where neighboring neurons in the source structure project to neighboring neurons in the target structure (Cang and Feldheim, 2013). The mechanisms involved in generating sensory maps may also be involved in the development of other systems (Cang and Feldheim, 2013). The mouse retinotopic map (Fig. 1) provides a model system to study topographic map formation, with an extensive range of mutant mice lines available (Frisén et al., 1998; Brown et al., 2000, 1998; Feldheim et al., 2000; Triplett et al., 2011). During development, axons from retinal ganglion cells (RGCs) grow through the optic tract to innervate the superior colliculus (SC). By postnatal day 1 (P1), RGC axons have grown all the way from the anterior to the posterior region of the SC, overshooting their final target locations (McLaughlin et al., 2003). The axons start branching, and then branches outside the topographically correct location are pruned away (McLaughlin et al., 2003). The map is topographically ordered before eye opening at P10–12 (McLaughlin et al., 2003), but the axonal arbor size continues to decrease for a few more weeks (Lyngholm et al., 2013).

Several candidate mechanisms have been proposed to guide RGC axons to their final locations:

1. The chemoaffinity hypothesis (Sperry, 1963; Meyer, 1998) which in its modern form has Ephs‐ and ephrins labelling orthogonal axes in the retina and SC (McLaughlin and O'Leary, 2005).
2. Activity‐dependent mechanisms driven by spontaneous retinal activity instructs map formation (Ackman et al., 2012), for example, Hebbian‐based modification of synaptic strengths (Willshaw and von der Malsburg, 1976).
3. Competition for resources (English et al., 2012) or space in the target tissue (Triplett et al., 2011; van Ooyen, 2011).
4. Partial mediolateral ordering of RGC axons within the optic tract (Plas et al., 2005).
5. Axon–axon interactions (Yates et al., 2004; Gebhardt et al., 2012).

The surface‐bound Eph receptors are tyrosine kinases and bind to members of the ephrin family of ligands, which are also surface bound (Cheng et al., 1995; Drescher et al., 1995). On binding, both cells can transduce a signal leading to changes in cellular behavior. EphA is expressed in an increasing nasal to temporal gradient in the retina, and ephrin‐A is expressed in an increasing anterior to posterior gradient in the SC. Stripe assay experiments show that growing axons bearing EphA are repelled by ephrin‐A substrates (Monschau et al., 1997). By selectively knocking out genes for individual types of Ephs and ephrins, the targeting in the colliculus is disrupted. For example, in *ephrin‐A2* ^−/−^mice, nasal injections in the retina yield one termination zone, whereas temporal injections yield ectopic projections with two termination zones. In contrast, *ephrin‐A5* ^−/−^yields ectopic projections for both nasal and temporal injections. EphB is expressed in an increasing dorsoventral gradient in the retina and ephrin‐B is expressed in an increasing mediolateral gradient in the SC. *In vivo* experiments in mouse show that knocking out EphB affects the direction of interstitial branching from the RGC axon shafts along the mediolateral axis, and suggests that the EphB–ephrin‐B interaction may be attractive (Hindges et al., 2002).

Insights from experiments with mutant mice gave rise to new computer models, several of which have been reviewed (Swindale, 1996; Goodhill and Richards, 1999; Goodhill and Xu, 2005; Goodhill, 2007). However, these reviews were qualitative and excluded recent genotypes (Cang et al., 2008; Triplett et al., 2011). We have created an open framework to compare model results quantitatively with experimental data and to compare models with each other.

We aimed to see if any model, under one set of parameter values, is consistent with all phenotypes. To make the task tractable, we reimplemented a representative subset of models (Whitelaw and Cowan, 1981; Gierer, 1983; Willshaw, 2006; Triplett et al., 2011) and applied them to phenotypes previously described in sufficient quantitative detail (Feldheim et al., 2000; Reber et al., 2004; Cang et al., 2008; Triplett et al., 2011). Key features of the resulting maps are quantified using virtual experiments and compared to experimental data. Our findings suggest that the models failed to account for the range of experimental data studied. Only one model can reproduce the collapse point seen in the *Isl2‐EphA3* ^ki/+^ phenotype, and two of the models fail to reproduce the *Math5* ^−/−^ phenotype. However, by reintroducing a weak gradient in the SC, the models can reproduce the global order still remaining in *ephrin‐A2,A3,A5* triple knock‐out (TKO) maps.

## METHODS

The modelling process had three main stages: (i) selection of mouse genotypes with retinotopic map data; (ii) selection of models from the literature to test against the data, and (iii) simulation of these models and comparison with the data. To enable a precise, quantitative comparison between different models and to generate the predictions, we simulated all models within the same modelling pipeline. The model pipeline had three phases comprising calculation of initial conditions, simulation of the development of connections, and analysis of the final connection patterns. All computer code and data relating to this project (pipeline, models, and analysis tools) are freely available (https://github.com/Hjorthmedh/RetinalMap).

### Genotype Selection

We used experiments from five mouse genotypes for which we believe there are sufficient quantitative data to constrain the models, and which are important in ruling out certain classes of model.

1. The most quantitative information comes from wild‐type mice, with both anatomical tracing data across development (Lyngholm et al., 2013), and whole maps acquired by intrinsic imaging data from adult mice (Cang et al., 2008).
2. and 3. The *Isl2‐EphA3* genotypes (heterozygous and homozygous knock‐in) disrupts the molecular positional information for around 40% of the RGCs by adding extra EphA3, providing phenotypes which allowed us to assess the impact of systematically modifying gradients on maps. The phenotypes from *Isl2‐EphA3* were characterized along projections from nasotemporal (NT) axis in the retina to the anteroposterior (AP) axis in the colliculus using retinal injections (Brown et al., 2000; Reber et al., 2004). Further combinations of *Isl2‐EphA3* with *EphA4* and *EphA5* knock‐outs (Reber et al., 2004; Bevins et al., 2011) were analyzed, but omitted here as results were qualitatively similar to earlier findings (Willshaw, 2006). The position of the collapse point (the point where the Isl2^+^ and Isl2^−^ maps merge) depended on the relative difference in EphA level between Isl2^+^ and Isl2^−^ cells. By knocking out *EphA4*, the relative difference increased, causing the collapse point in *Isl2‐EphA3* ^ki/+^ to move temporally. For combined *Isl2‐EphA3* ^ki/+^ *EphA5* mutants, the effect was similar, with the homozygous knock‐out of *EphA5* moving the collapse point further temporally than the heterozygous knock‐out.
3. In TKO of *ephrin‐A2,A3,A5*, all the ephrin‐As participating in map formation along the AP axis of the SC were removed. The whole map was characterized by intrinsic imaging (Cang et al., 2008) and analyzed using the Lattice method (Willshaw et al., 2014).
4. The *Math5* ^−/−^ knock‐out has a reduced RGC population in the retina, reducing competition between RGC axons. The phenotype has been characterized mainly by whole eye injections that give the density of the SC projections (Triplett et al., 2011).

Many other mutant mice lines have been characterized by antereograde or retrograde labelling of axons, including knockouts of ephrin‐A2 and ephrin‐A5 (Feldheim et al., 2000) and EphA7 (Rashid et al., 2005). This data was more challenging to quantify as (i) there was one injection site per individual and (ii) there appeared to be considerable variation in the locations of termination zones between individuals (Feldheim et al., 2000). The variability meant, it was not possible to create a single composite map (as in the case of the *Isl2‐EphA3* knock‐ins) from multiple individuals. We, therefore, decided to exclude these data from this quantitative comparison. We also excluded mutant mice lines that perturbed retinal activity (e.g., *β*2^−/−^; McLaughlin et al., 2003) as two of the models studied here excluded activity‐dependent mechanisms.

### Choice of Models

The main criteria used for our choice were that (i) the models contained mechanisms that provided flexibility in the pattern of connections formed; (ii) the models simulated the development of two‐dimensional maps, or could be extended to do so; and (iii) they had explicit representations of gradients to allow manipulations in gradients to be simulated.

1. Prestige and Willshaw (1975) suggested a classification of the different ways in which graded labels could instruct retinotopic mappings. In Type I mechanisms, the gradients provide each cell with a preferred location that matches the topographically correct position (Sperry, 1963; Meyer, 1998). In Type II mechanisms, all axons prefer the same location, but with different affinity (Prestige and Willshaw, 1975). Together with a competition mechanism, the map then organizes itself so that the RGC with highest affinity for the location with highest affinity innervates it, leaving the RGC with the next greatest affinity to innervate the SC neuron with the next greatest affinity, and so on. Type I models establish connections by matching up fixed‐value labels on RGC axons with those on SC neurons. In the *Isl2‐EphA3* mutant maps, the abnormally high values of EphA in much of the retina have no counterpart in the colliculus, yet all the retina projects to the colliculus. This finding rules out strict Type I models.
2. We excluded the 1D branching model by Yates et al. (2004) as we were unable to make a 2D model from the information provided.
3. We also excluded the model of Simpson and Goodhill (2011), as chemoaffinity is represented implicitly by a term describing the distance of an axon from its correct location, and the model by Grimbert and Cang (2012), as no method was given to convert gradients to the probability maps used in their simulations (Sterratt and Hjorth, 2013).

We selected four models that included a range of developmental mechanisms implicated in the development of retinotopic maps. (Sterratt and Hjorth, 2013). Here, we refer to the models by the surname of either the first author of the relevant publication or the principal architect. We chose the following models:

1. The Gierer (1983) model exists as both Types I and II versions, the Type II version including a mechanism akin to competition. Here, we use an updated version of Gierer's Type II model (Sterratt, 2013) in which the strength of competition can be modified.
2. The Koulakov model (Triplett et al., 2011), which builds on a series of models from Koulakov and coworkers (2004, 2006, 2010), is a generalization of the Gierer model including an abstract representation of correlated retinal activity.
3. The Whitelaw model (Whitelaw and Cowan, 1981) combines a Hebbian activity scheme (Willshaw and von der Malsburg, 1976) with a Type II affinity mechanism. It has an explicit representation of retinal activity and a multiplicative interaction between activity and gradients.
4. The Willshaw model (von der Malsburg and Willshaw, 1977; Willshaw, 2006), also known as the “Marker Induction model,” uses a Type I gradient matching scheme where the SC gradients are modifiable during development by the action of the incoming retinal fibers.

The Gierer, Whitelaw, and Willshaw models were proposed before the discovery of Ephs and ephrins (von der Malsburg and Willshaw, 1977; Whitelaw and Cowan, 1981; Gierer, 1983). In later versions of both the Gierer model and the Willshaw model, the specifics of these graded labels were introduced (Willshaw, 2006; Sterratt, 2013). Here, we have made additional extensions to make all models 2D. In all cases, a single molecule type (A or B) labels each axis of the retina and the SC. The Gierer model has spatially restricted sprouting, such that new synapses are generated close to existing ones [as did the original Willshaw model (von der Malsburg and Willshaw, 1977)]; in the other models, new synapses can be placed with fewer constraints in the SC, irrespective of the location of previous synapses.

Whenever possible, we used the model parameters as described in the original paper. One parameter was changed for the Willshaw model, while the Koulakov model required us to rebalance the values of the neural activity and the chemical cue strength. The Gierer and the Whitelaw models had their equations modified, however, we retained the original values of the parameters and manually adjusted a selected few parameters to give the desired behavior (Table 1).

**Table 1:** Parameter Values Used in the Models

Parameter	Default Value	Original Value	Meaning
*General*			
*N*_R_	2000	n/a	Number of RGCs
*N*_SC_	2000	n/a	Number of SC neurons
*d*_R_	0.0139	n/a	Exclusion distance in retina
*d*_SC_	0.0119	n/a	Exclusion distance in SC
*Gierer*			
*N*_term_	16	16	Number of terminals made by each RGC
*ε*	0.005	0.005	Growth rate for competition
*η*	0.1	n/a	Decay rate for competition
*Koulakov*			
*α*	90	20	Chemical strength of A‐system
*β*	135	30	Chemical strength of B‐system
*γ*	5/16	1/20	Strength of activity interaction
*b*	0.11	0.11	Retinal correlation distance
*a*	0.03	0.03	SC interaction distance
*Whitelaw*			
*r*_R_	0.07	n/a	Radius of retinal activity
*r*_SC_	0.0289	n/a	Radius of SC interaction
*µ*	0.1	0.1	Weight decay rate
Δ*t*	0.0001	[0.05, 0.5]	Integration time step
*w*_min_	0.00001	0.009	Minimum synapse strength
*Willshaw*			
*σ*	0.05	0.05	Induced marker source strength
*δ*	0.01	0.01	Induced marker diffusion strength
*θ*	0.1	0.1	Speed of weight update
*κ*	0.0504	0.0504	Sharpness of receptor‐ligand comparison
*ζ*	1	3.5	Scale of induced marker and ligand interaction
Δ*t*	1	0.1	Integration time step
*w*_min_	0.001	n/a	Minimum synapse strength

Column 2 denotes the parameter values used in this study, compared to those used in previous studies (column 3).

### Pipeline Phase 1—Initialization

The positions of neurons in retina and SC and the concentration of EphA/B receptors and ephrin‐A/B ligands define the initial conditions of the simulations for the different genotypes. These can then be passed to one of the models defined below, and the retinotopic map formation simulated. The initial connections set up by each model are described in the relevant sections below and summarized in Table 2.

**Table 2:** Initial Conditions for the Four Models

Model	Initial Connectivity
Gierer	Each RGC connected to 16 random SC neurons
Koulakov	No connections
Whitelaw	Fully connected, with weight 1
Willshaw	Fully connected, weights uniformly sampled from [0, 10^–4^]

#### Number and Placement of Neurons

In mouse, there are around 50,000 RGCs (Jeon et al., 1998; Salinas‐Navarro et al., 2009), and an unknown number of SC neurons. Here, networks containing 2,000 retinal neurons (*N*_R_) and 2,000 SC neurons (*N*_SC_), were simulated. We believe these populations to be large enough to represent the system, without being too large to make the models too demanding in computation time. The positions of neurons were drawn randomly from a uniform 2D distribution (Galli‐Resta et al., 1997; Eglen, 2012). If there were no other neurons within a certain specified distance, this position was accepted. The algorithm terminated when the required number or neurons *N*, had been placed within the structure, or 1,000 *N* positions had been rejected in total. To minimize edge effects, neurons were also placed outside the target structure, but were not counted in the final population. This prevented an artificial inflation of the density of neurons at the edges. The retinal size was normalized to unit size and the retinal neurons were placed within a circle of diameter 1. The shape of the SC (Fig. 1) was taken from Figure 2 in Dräger and Hubel (1976). The minimum distance was set separately for retina (*d*_R_) and SC (*d*_SC_) so that 2,000 neurons would fit inside the space available (Table 1).

**Figure 2:**
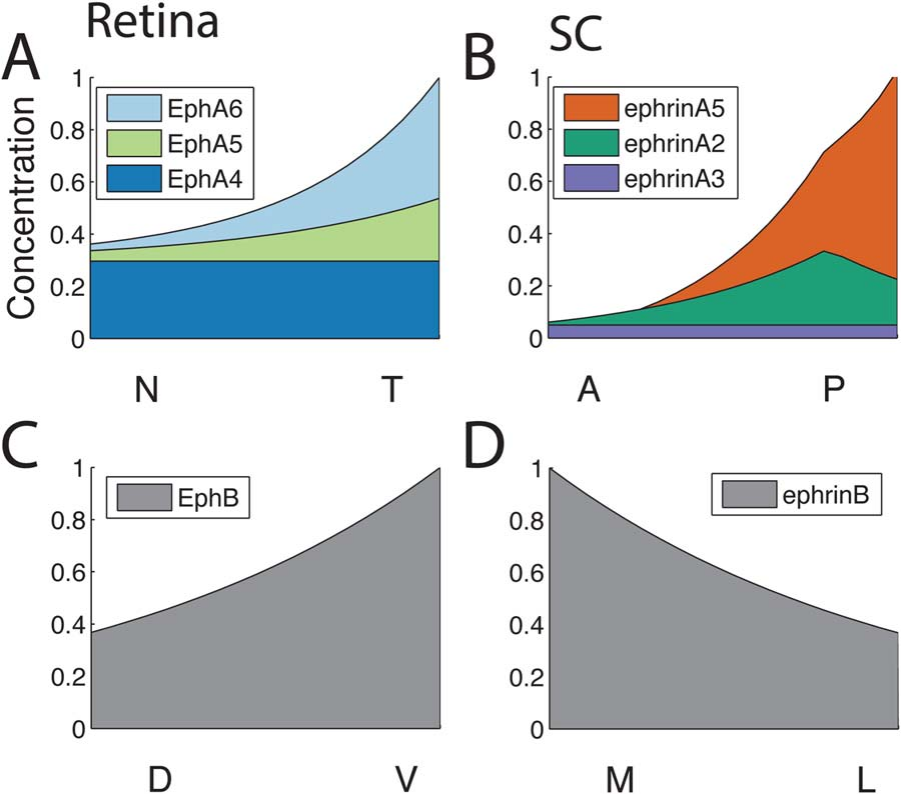
Wild type Eph and ephrin gradients used in the model comparison. (A) Retinal EphA gradients, (B) SC ephrin‐A gradients, (C) Retinal EphB gradients, and (D) SC ephrin‐B gradients. Parameters for the gradients are given in Table 3.

#### Specifying Gradients

Despite their importance for map formation (McLaughlin and O'Leary, 2005), the only quantitative measures of Eph/ephrin gradients is for retinal EphA where mRNA levels were measured at P1 using *in situ* hybridization along the NT axis (Reber et al., 2004) and modelled as shallow exponential gradients. By contrast, there is no quantitative data for ephrin‐As or the B‐system (Hindges et al., 2002) and so we have assumed exponential profiles. In parallel with these forward gradients, there is a second set of opposing countergradients of Eph receptors in the SC and ephrin ligands in the retina (Fig. 1). These countergradients have not been quantified in either the retina or the SC. Since recent work showed that countergradients can be replaced by a competitive mechanism that enforces an even distribution of synapses for each retinal neuron (Sterratt, 2013), we have focused on the forward system and excluded countergradients. Table 3 describes how we have quantified the gradients, which are displayed pictorially in Figure 2. The gradients are identical for all repeats of a given genotype, but they are sampled at the neuron locations, which vary between runs. We assume the affinities of the receptor subtypes are similar and the individual gradients are summed to give the total expression of EphA, EphB, ephrin‐A, and ephrin‐B at any point in retina and SC (Brown et al., 2000; Bevins et al., 2011).

**Table 3:** Quantitative Representation of Eph and ephrin Gradients in RGCs and SC

Protein	*G*0	*G*1	*G*2	*G*3	Source
*Retinal Eph gradients*					
EphA4	1.05	0	0	1	Measured (Reber et al., 2004)
EphA5	0	0.85	1.8	1	Measured (Reber et al., 2004)
EphA6	0	1.64	2.9	1	Measured (Reber et al., 2004)
EphA3 from *Isl2‐EphA3* ^ki/ki^	1.86	0	0	1	Measured (Reber et al., 2004)
EphA3 from *Isl2‐EphA3* ^ki/+^	0.93	0	0	1	Measured (Reber et al., 2004)
EphB	0	1	1	1	Postulated (McLaughlin and O'Leary, 2005)
*SC ephrin gradients*					
ephrin‐A2	−0.06	0.35	2	0.8	Estimated (Frisén et al., 1998; Feldheim et al., 2000; Hansen et al., 2004)
ephrin‐A3	0.05	0	0	1	Estimated (Pfeiffenberger et al., 2006; Triplett et al., 2012)
ephrin‐A5	−0.1	0.9	3	1	Estimated (Frisén et al., 1998; Feldheim et al., 2000; Hansen et al., 2004; Rashid et al., 2005)
ephrin‐B	0	1	1	0	Postulated (McLaughlin and O'Leary, 2005)

Retinal EphA gradients were measured (Reber et al., 2004); “estimated” values are our measurements from published figures; “postulated” means gradients have been proposed based on limited data. The gradient at a point *x* is given by *G*(*x*) = max(0, *G*_0_ + *G*_1_ exp(−*G*_2_|*x* − *G*_3_|)) where *x* *∈* [0, 1] is the position along an axis (NT, dorsoventral, AP or mediolateral). The gradients of each subtype are summed together. The summed gradients were normalised such that the peak value for each of the summed WT gradients were 1. This scaling was kept for all phenotypes. Thus, for EphA3 knock‐ins, the peak gradient was larger than 1, and for knock‐out phenotypes the peak gradient was less than 1.

To explore the effect of a weak signalling molecule, for *ephrin‐A2,A3,A5* TKO, we introduced a weak gradient running along the rostrocaudal axis of the SC with the same shape as the ephrin‐A gradient assumed for the wild type but with strength multiplied by a constant *K <* 1 to scale it down.

#### Model Configuration

To ensure a fair comparison, all models were created with the same spatial geometry in retina and SC. The number of neurons in retina and SC was also fixed, and neurons were positioned according to the minimal spacing rules described earlier (for parameters see Table 1, top four rows). All models were restricted to use one set of parameter values for all genotypes (Table 1, remaining rows). The parameter values in three models were optimized manually to fit one experimental condition (Gierer was optimized for *Math5* ^−/−^, Koulakov for *Isl2‐EphA3* ^ki/+^, and Whitelaw for *Isl2‐EphA3* ^ki/ki^). The Willshaw model did not require any additional parameter tuning beyond that presented in 2006.

### Pipeline Phase II—Running the Simulations

Models written in MATLAB, R, and C have been integrated into the pipeline. Implementation details of each model are described later. Each genotype was run 10 times with different initial conditions (positions of neurons, gradients, and initial connectivity) for each model, to assess the variability of the simulated results.

### Pipeline Phase III—Analysis

The aim is to perform an unbiased comparison of model results and experiments using appropriate quantitative measures. We have assembled a set of measures for analyzing both simulated maps and those from experimental recordings.

#### Discrete Versus Continuous Synapses

All models represent the map as a set of connections in a weight matrix. Two of the models use discrete (integer‐valued) weights. For the other two models, which use continuous valued connections, some of the measures require the weights below certain small values to be set to zero; these thresholds are given in Table 1.

#### Map Precision

This has been measured in developing mouse by dual retrograde injections (Lyngholm et al., 2013). Two injections of red and green beads marked two groups of neurons in SC and the label was retrogradely transported to RGCs. The spatial segregation of the two labelled RGC populations was then assessed (Upton et al., 2007; Lyngholm et al., 2013). The segregation measure is defined as the fraction of RGCs where the nearest neighbor is the same color. For two completely segregated projections, the value is 1; for two overlapping projections, the value is 0.5. Here, we performed equivalent virtual injections on simulated maps to assess map precision.

#### Contour Analysis

The distribution of synaptic labelling in the retina following dye injection in the SC was assessed using contour analysis based on kernel density estimates. Gaussian kernels were placed around a set of discrete labelled points to estimate the variations in density throughout the region. The retinal space was divided into a 100 × 100 grid, and each labelled point had the same weight. The kernel density estimate at location **r** in the grid was defined by

(1) $$\hat{f}(r,k)=\frac{1}{N}\frac{1}{2\pi k^{2}}\overset{N}{\underset{i=1}{\sum }}\text{exp}\left(-\frac{1}{2k^{2}}|r-r_{i}{|}^{2}\right)$$

where **r**_*i*_ is the locations of the *N* labelled neurons and the bandwidth *k* is chosen (using fminsearch in MATLAB) to maximize the cross‐validated log‐likelihood

(2) $$L(k)=\overset{N}{\underset{i=1}{\sum }}\text{log}({\hat{f}}_{i}(r_{i},k))$$

Where *f*^*_i_* (**r**, *k*) is the kernel density estimate with data point *i* excluded. The contour curves were defined so that, for example, the 25% contour encloses the top 25% percentile of the total labelling from the kernel density estimate (Sterratt et al., 2013). The readout is the retinal area covered by the respective contour curve.

#### Lattice Method Analysis

The Lattice method (Willshaw et al., 2014) allows the quality, orientation and precision of point‐to‐point maps to be quantified. It has been applied to maps measured by simultaneous visual field stimulation and Fourier‐based intrinsic imaging of mouse SC (Cang et al., 2008). The method operates on pairs of matched points located in visual field and SC. In the experiments, each of the 62,500 pixels represents a point location in the SC. For each pixel, the matching point in the visual field is the one where stimulation excites the pixel maximally.

In the first step of the method when applied to experimental data, approximately 150 visual field points are chosen to be centers. These are spaced approximately equidistantly, the separation being limited by the resolution of the Fourier method. Associated with each center point is the group of points lying within a small circle around it. The radius of the circle is chosen as half the separation between nearest neighbors to ensure maximum coverage of the visual field while keeping the overlap between circles small. The 150 corresponding nodes in the SC are determined by the centroids of the projection patterns from the points surrounding each field center. Delaunay triangulation is then used to construct a lattice on the field nodes, and the edges of this lattice are then projected into the SC. Edges that cross in the map in the SC indicate local map distortions. Connected nodes are then removed one by one to form the largest ordered SC submap in which no edges cross. The numbers of nodes and edges remaining within the largest ordered submap are indicators of the overall map quality. To give an overall measure of the orientation of the SC map relative to the field, the mean difference in orientations of corresponding edges in the field and the SC is computed.

To apply the Lattice method to mappings from simulations, we took the points in the retina to be the set of 2,000 RGC locations **r**_*i*_. For each RGC *i*, the corresponding SC neuron *j*, located at **s**_*j*_, was the one with the strongest connection strength *W_ij_* from *i*. The Lattice method was then applied to this set of paired points, but with 100 rather than 150 center nodes, and with the radius of circles in the retina being 7% of the retinal diameter. This reduction in node number was necessary due to the smaller number of points in the simulations (2,000) than the experiments (62,500); even so there was some overlap of the points within the circles of neighboring centers for the modelled data. Over different simulations, the average number of times that a single point was used varied between 1.7 and 2.2.

To assess the global order along the AP axis, we computed the AP polarity, which is defined as the percentage of neighboring node pairs in the lattice that are in the correct AP order relative to each other, given their positions on the NT axis. The equivalent mediolateral polarity was also calculated.

#### Visualizing Projections and Collapse points

In the *Isl2‐EphA3* ^ki/+^ mutant, anterograde injections in nasal retina resulted in two separated termination zones, whereas a temporal injection gave one termination zone, see Figure 4(B,H) in Brown et al. (2000). These authors plotted the locations of anterograde injections of dye along the NT retinal axis against the locations of the termination zones along the AP axis of the SC. In Reber et al. (2004), this experimental paradigm was extended. The position where the two maps converge into one was termed the collapse point. We have automated the collapse point detection. The NT axis was divided into 50 equal‐sized bins, and the projections originating from each bin were clustered separately based on their termination points using the *k*‐means algorithm. If the distance between the means of the two clusters in the SC was larger than 1.5 standard deviations and the smaller of the clusters contained at least 5% of the neurons, then the two clusters were considered distinct. The algorithm defined the nasal‐most bin with only one cluster as the collapse point. In some of the cases studied, there was no collapse point present.

### Models

Here, we describe the mechanisms of each model, listing its key features and how they were adapted for this study. We describe all models in the same notation, which in some cases required a change in notation from the published version.

### Gierer Model

The Gierer model (Gierer, 1983; Sterratt, 2013) is a relatively simple model of map formation that was originally formulated in 1D and incorporates both gradients and countergradients, which are used to define a potential that guides where synapses are placed. The model has been extended to 2D with the countergradients removed. The competition term also has an added decay term to prevent it from growing without bound (Nissenbaum, 2010).

Each RGC axon has *N*_term_ = 16 terminals. One epoch, equivalent to advancing time by one step, consists of examining each terminal in the system and deciding whether to move it. Each terminal is considered sequentially in random order. For a terminal that connects retinal neuron *i*, with retinal coordinates **r**_*i*_, to SC neuron *j*, with SC coordinates **s**_*j*_, the terminal can move to one of the neighbors *j′* of *j* (neighbors defined by the Delaunay triangulation on the *N*_SC_ SC neurons) which has lowest potential. The potential at location *j* is

(3) $$p(r_{i},s_{j^{\prime }})=g(r_{i},s_{j^{\prime }})+c(s_{j^{\prime }})$$

where *g*(·,·) is the gradient interaction defined below and *c*(**s**_*j′*_) is the level of competition at point **s**_*j′*_ in the SC. Designating cell *j* *∗* as the neighbor with the lowest potential, the terminal moves to cell *j* *∗* if this potential is lower than the potential at its original position *j* (i.e., *p*(**r**_*i*_, **s**_*j*_ *∗* *) < p*(**r**_*i*_, **s** *_j_*)).

#### Gradient Term

(4) $$g(r_{i},s_{j})=R_{A}(r_{i})L_{A}(s_{j})-R_{B}(r_{i})L_{B}(s_{j})$$

Here, *R*_A_ and *R*_B_ are the retinal EphA and EphB receptor concentrations, and *L*_A_ and *L*_B_ are the SC ephrin‐A and ephrin‐B concentrations. A RGC axon with a high level of EphA is more sensitive to ephrin‐A in the SC than an axon with a lower level of EphA. The difference in signs of the two terms indicates that A is a repulsive system since the potential increases with increased concentrations, whereas B is an attractive system where the potential instead decreases with increased concentrations.

#### Competition Term

Competition was introduced by incorporating the term *c*(**s** *_j_*) which grows at a rate *ρ*(**s** *_j_*), the density of terminals contacting on SC neuron *j* (Gierer, 1983). This term ensures an even distribution of connections over the SC. However, this assumes infinite memory, with the value of *c* increasing without bound. Following recent analysis (Sterratt, 2013), the decay term *ηc*(**s** *_j_*) was added to weaken competition by removing the infinite memory

(5) $$\frac{\partial c(s_{j})}{\partial t}=\epsilon \rho (s_{j})-\eta c(s_{j})$$

To check for a steady‐state in the network, we compared the values of *c* with their theoretical steady‐state, *c*(**s** *_j_*) = (ϵ/*η*)*ρ*(**s** *_j_*). Simulations verified that the maps had converged after 10,000 epochs.

There are three key parameters in the model. *N*_term_ was fixed at 16, following Gierer (1983). A small value of the compensation factor *ε* was chosen to ensure that competition was gradually enforced. The value of *η* was then chosen so that the competition term was relatively weak in the *Math5* ^−/−^ condition. Its effect was 10 times stronger in wild type, as *Math5* ^−/−^ has 10% of RGCs compared to wild type.

#### Summary of Modifications

Our implementation of the Gierer model has bounded competition, and no countergradients.

### Koulakov Model

The Koulakov model (Triplett et al., 2011) uses gradient information, competition, and correlated retinal activity to define a system energy for the current set of connections. The system evolves by repeatedly modifying connections, favoring modifications that reduce energy. In the Koulakov model, the energy of the system consists of three terms, representing the interaction of the chemical cues, the effect of correlated neural activity and the effect of competition for resources

(6) $$E=E_{\text{chem}}+E_{\text{act}}+E_{\text{comp}}$$

The chemical energy represents the repulsive interaction between EphA and ephrin‐A and the attractive interaction between EphB and ephrin‐B.

(7) $$E_{\text{chem}}=\underset{i\in \text{synapses}}{\sum }\left(\alpha R_{A}\left(r_{\mu_{i}}\right)L_{A}\left(s_{\mu_{i}}\right)-\beta R_{B}\left(r_{\nu_{i}}\right)L_{B}\left(s_{\nu_{i}}\right)\right)$$

where *α* and *β* define the relative strengths of the A and B system, *R*_A_ and *R*_B_ are the receptor concentrations for RGC at **r**, and *L*_A_ and *L*_B_ are the ligand concentrations for SC neuron at **s**; *µ_i_*, *ν_i_* map synapse *i* onto its corresponding RGC and SC neuron index.

The neural activity term represents the influence of correlated activity on the synapses (Tsigankov and Koulakov, 2006)

(8) $$E_{\text{act}}=-\frac{\gamma }{2}\underset{i,j\in \text{synapses}}{\sum }C\left(r_{\mu_{i}},r_{\mu_{j}}\right)U\left(s_{\nu_{i}},s_{\nu_{j}}\right)$$

where *C* represents the retinal correlation and *U* the pairwise interaction in the SC

(9) $$C(r_{\mu_{i}},r_{\mu_{j}})=\text{exp}(-|r_{\mu_{i}}\;-r_{\mu_{j}}|/b)$$

(10) $$U(s_{\nu_{i}},s_{\nu_{j}})=\text{exp}(-{\left(s_{\nu_{i}}\;-s_{\nu_{j}}\right)}^{2}/2a^{2})$$

where *b* and *a* specify the space constants. The competition term provides an initial drive to add synapses, but also limits the total number of synapses in the system. It is defined as

(11) $$E_{\text{comp}}=\underset{i\in \text{RGCs}}{\sum }\left(-500n_{R,i}^{0.5}+n_{R,i}^{2}\right)+\underset{j\in \text{SC}\;\text{cells}}{\sum }n_{SC,j}^{2}$$

where *n_R_*_,*i*_ and *n_SC_*_,*j*_ are the number of synapses made by RGC *i* and SC neuron *j*. Here *i* is summed over all RGCs and *j* over all SC neurons. The model starts without any synapses. With a small number of synapses, initially *E*comp is negative (Term 1), reducing the total energy and favoring connection formation. As the number of synapses increases *E*comp grows positive making connection formation more difficult.

Each iteration of the model has two steps. First, the algorithm attempts to add a connection between a randomly chosen pair of RGC and SC neurons. In the second step, the algorithm tries to remove a randomly chosen existing connection. In both cases, a change is accepted with probability *p* = 1/(1 + exp(4Δ*E*)), where Δ*E* is the change in energy associated with adding or removing the synapse. This means that changes that increase the energy are unlikely to be accepted.

#### Summary of Modifications

The original model parameters have been adjusted to better account for the *Isl2‐EphA3* ^ki/+^ phenotype: the chemical strength (*E*_chem_) was multiplied by a factor of 4.5, and the neural activity (*E*_act_) was multiplied by a factor of 0.8 (Table 1). The activity term (*E*_act_) was then multiplied by a factor of 5 to compensate for the reduced number of synaptic pairs when the number of neurons was reduced to 2,000 from 10,000, the value used in Triplett et al. (2011).

Model convergence was assessed by tracking the average spread of postsynaptic connections in the SC for the RGC axons, or by tracking the fraction of rejected actions, which grows as the model gets closer to convergence. To ensure convergence, each simulation was run for 10,000 epochs. The number of iterations in an epoch is equal to the number of neurons in the simulated retina or SC, so that on average each neuron will have had an addition and a removal step per epoch. The total number of iterations was, thus, 2,000 *×* 10,000.

### Whitelaw Model

The Whitelaw model (Whitelaw and Cowan, 1981) uses chemical cues and explicit retinal activity patterns to adapt synaptic weights in a Hebbian fashion. The strength of the connection between RGC *i* with location **r**_*i*_ and SC neuron *j* (location **s**_*j*_) is represented by *W_ij_*. The system starts fully connected with all weights initialized to 1.

Chemospecificity is introduced through adhesive coefficients *M_ij_* between RGC *i* and SC neuron *j*. Mimicking the expression for chemospecificity in the original model, we define *M_ij_* as

(12) $$M_{ij}=R_{A}(r_{i})\left[\underset{k}{\text{max}}(L_{A}(s_{k}))-L_{A}(s_{j})\right]+R_{B}(r_{i})L_{B}(s_{j})$$

Compared to the original formulation (Whitelaw and Cowan, 1981), the contribution of the A system has been altered to make it repulsive and to ensure that the adhesive coefficients remain positive, which is needed for synaptic plasticity [Eq. (16)]. The B system is attractive, as was assumed for the markers in the original 1D system (Whitelaw and Cowan, 1981).

Retinal waves are modelled by activating RGCs within a circular region. For each RGC *i* and SC neuron *j*, the set neigh(**r** *_i_*) contains the indices of RGCs falling within a radius *r*_R_ of **r** *_i_* (including *i* itself). The set neigh(**s** *_j_*) was defined similarly with a radius *r*SC on the SC.

The algorithm proceeds on an epoch basis. For *q* *∈* 1,..., *N_R_*, RGC *q* is the center of activity and retinal activities, *x_i_*, are set using

(13) $$x_{i}=\{\begin{array}{cc} u & i\in \text{neigh}(r_{q}),\text{where}\;u=2/|\text{neigh}(r_{q})|\\ 0 & \text{otherwise} \end{array}$$

This normalizes the sum of RGC activity to 2, removing small spatial variations in the density of neurons that otherwise affect topography. This reflects the formulation in the original model where the induced activity in the SC was scaled to be smaller than the activity input in the retina (Whitelaw and Cowan, 1981).

The induced activity in SC neuron *j* is denoted by *y_j_^I^*

(14) $$y_{j}^{I}=\overset{N_{r}}{\underset{i=1}{\sum }}W_{ij}x_{i}$$

Each SC neuron receives lateral input from other SC neurons within a radius *r*_SC_.

(15) $$y_{j}=\frac{k}{\left|\text{neigh}(s_{j})\right|}\underset{p\in \text{neigh}(s_{j})}{\sum }y_{p}^{I}$$

where *k* is a proportionality constant retained from the original model.

The Hebbian change in the weight matrix *W_ij_* resulting from RGC *q* being the center of activity is given by

(16) $$\Delta W_{ij}^{q}=\Delta t\left(\left(M_{ij}+1\right)x_{i}y_{j}-\mu y_{j}\right)$$

where Δ*t* is the time step per activation in the retina, *M_ij_* is the chemospecific adhesion [Eq. (12)] and *µ* is the rate at which synapses decay due to asynchronous activity. The addition of 1 to *M_ij_* reflects the original model's baseline gradient value, which aims to ensure that when RGC *i* and SC cell *j* are coactive the change to the synapse strength is positive.

The total change in *W_ij_* over an epoch is $\Delta W_{ij}=\sum_{q}\Delta W_{ij}^{q}$. At the end of an epoch, *W_ij_* is updated

(17) $$W_{ij}(t+1)=W_{ij}(t)+\Delta W_{ij}.$$

Any elements in *W_ij_* below a small threshold value *w*_min_ were set to zero. Competition is introduced to prevent unbounded growth by normalizing the matrix *W* at the end of each epoch. The normalization is first done for each SC neuron, and then for each RGC

(18) $$W_{ij}\leftarrow \frac{N_{R}W_{ij}}{\sum_{i}W_{ij}}\;,\;W_{ij}\leftarrow \frac{N_{SC}W_{ij}}{\sum_{j}W_{ij}}.$$

This order of normalization is crucial for the formation of a double map in the *Isl2‐EphA3* ^ki/ki^ phenotype: normalizing first along inputs to SC neurons maintains the effect of the different levels of EphA (which affect the growth rate of connections) in the input RGCs. Reversing the order of normalization would remove the effect of the knock‐in.

#### Summary of Modifications

The Whitelaw and Cowan (1981) model was extended from 1D to 2D. The chemospecificity term now contains one attractive and one repulsive gradient. The retinal waves were changed to activate neurons within a radius *r*_R_ and the total retinal activity were normalized to maintain a constant level of activation for each wave. The weights were normalized after each epoch instead of after each activation. The number of neurons was increased from 20 to 2,000. The model parameters were optimized to fit the *Isl2‐EphA3* ^ki/ki^ data, which requires that postsynaptic normalization is done before presynaptic normalization.

### Willshaw Model

The key concept in the Willshaw (2006) model is that SC gradients are not fixed, but are “induced” by ingrowing retinal fibers. Each RGC *i* bears fixed quantities of EphA and EphB depending on retinal position according to the standard gradients. Levels of induced marker *I_j_* ^A^, *I_j_* ^B^ in SC neuron *j* depend on the densities of receptor in the terminals of the axons impinging on it, weighted by the appropriate synaptic strengths

(19) $$I_{j}^{A}=\underset{k}{\sum }W_{kj}R_{A}(r_{k})/\underset{k}{\sum }W_{kj}\;,\;I_{j}^{B}=\underset{k}{\sum }W_{kj}R_{B}(r_{k})/\underset{k}{\sum }W_{kj}.$$

The markers *T_j_* ^A^ and *T_j_* ^B^ represent the densities of the ligands ephrin‐A and ephrin‐B in each SC neuron. Unlike *L*_A_ and *L*_B_ in the other models, *T* ^A^ and *T* ^B^ vary over time, and are produced at a rate which depends on the relationship of the induced marker and the ligand

(20) $$\Delta T_{j}^{A}=(\sigma (1-\zeta I_{j}^{A}T_{j}^{A})+\delta \nabla^{2}T_{j}^{A})\Delta t\;,\;\Delta T_{j}^{B}=(\sigma (I_{j}^{B}-T_{j}^{B})+\delta \nabla^{2}T_{j}^{B})\Delta t$$

where *σ*, *ζ*, and *δ* are parameters and Δ*t* is the time step [set equal to 1 in Willshaw (2006)]. The parameter *ζ* is the sole modification to the model. It is set to 3.5 to compensate for the different magnitude of the wild type EphA gradients in the pipeline (maximum of 1) compared to the original model (maximum of circa 3.5). The Laplacian operator ▿^2^ enforces spatial continuity through short range interchange of markers between neuron *j* and its neighbors, which are defined by the links in a Delaunay triangulation of the SC neuron locations, where edges making angles smaller than 10° have been removed. Each synaptic connection is updated according to the similarity Φ_*ij*_ between the axonal and SC neuron markers, and a presynaptic competitive normalization

(21) $$\Phi_{ij}=\text{exp}\left(-\left[{\left(\zeta R_{A}(r_{i})T_{j}^{A}-1\right)}^{2}+{\left(R_{B}(r_{i})-T_{j}^{B}\right)}^{2}\right]/2\kappa^{2}\right)$$

(22) $$\Delta W_{ij}=\left(W_{ij}+\theta \Delta t\Phi_{ij}\right)/\underset{k}{\sum }\left(W_{ik}+\theta \Delta t\Phi_{ik}\right)-W_{ij}$$

Examination of Willshaw's code showed that *κ* = 0.0504 rather than the *κ* = 0.72 reported. Simulations were run with Δ*t* = 0.1 for 48,000 steps. Some long simulations (1,200,000 steps) were also run to investigate the stability of the maps. To set up the polarity, the model requires either a weak bias in the initial weights, or a weak bias in the gradients; here the latter was used and the initial connection weights were sampled from the uniform distribution. To initialize the simulation, each synaptic strength *Wij* is drawn independently from a uniform distribution between 0 and 10^−4^. The initial SC ephrins were taken from the standard gradients, that is, *T* ^A^ = *L*_A_(**s** *_j_*). These gradients are of a similar magnitude to those used in the original model (Willshaw, 2006), though with no noise.

#### Summary of Modifications

The gradients were taken from Table 3, meaning that the EphA gradient was a scaled‐down version of that used in Willshaw (2006) and the other gradients differed in form from the original ones, though were of a similar scale. Furthermore, in Willshaw (2006) noise was added to the gradients, which was not present here. The parameter values used (Table 1) were the same as those used in Figure 7 of Willshaw (2006), apart from *ζ*, which was adjusted to compensate for the scaling down of the retinal EphA gradient.

## RESULTS

By implementing four models and integrating them into our model evaluation pipeline (described in detail in Methods section), we could compare quantitatively each model's ability to account for each phenotype. The models received similar initial conditions for neuronal position and gradients (Fig. 2), while the pattern of initial connectivity was set according to each model. The resulting connectivity maps were evaluated using the same criteria for all models, thus ensuring a fair comparison. The four models were the Gierer model (Gierer, 1983; Sterratt, 2013), the Koulakov model (Triplett et al., 2011), the Whitelaw model (Whitelaw and Cowan, 1981), and the Willshaw model (Willshaw, 2006). For a detailed description of each model and why it was chosen, see Methods section.

### Wild Type

**Figure 3:**
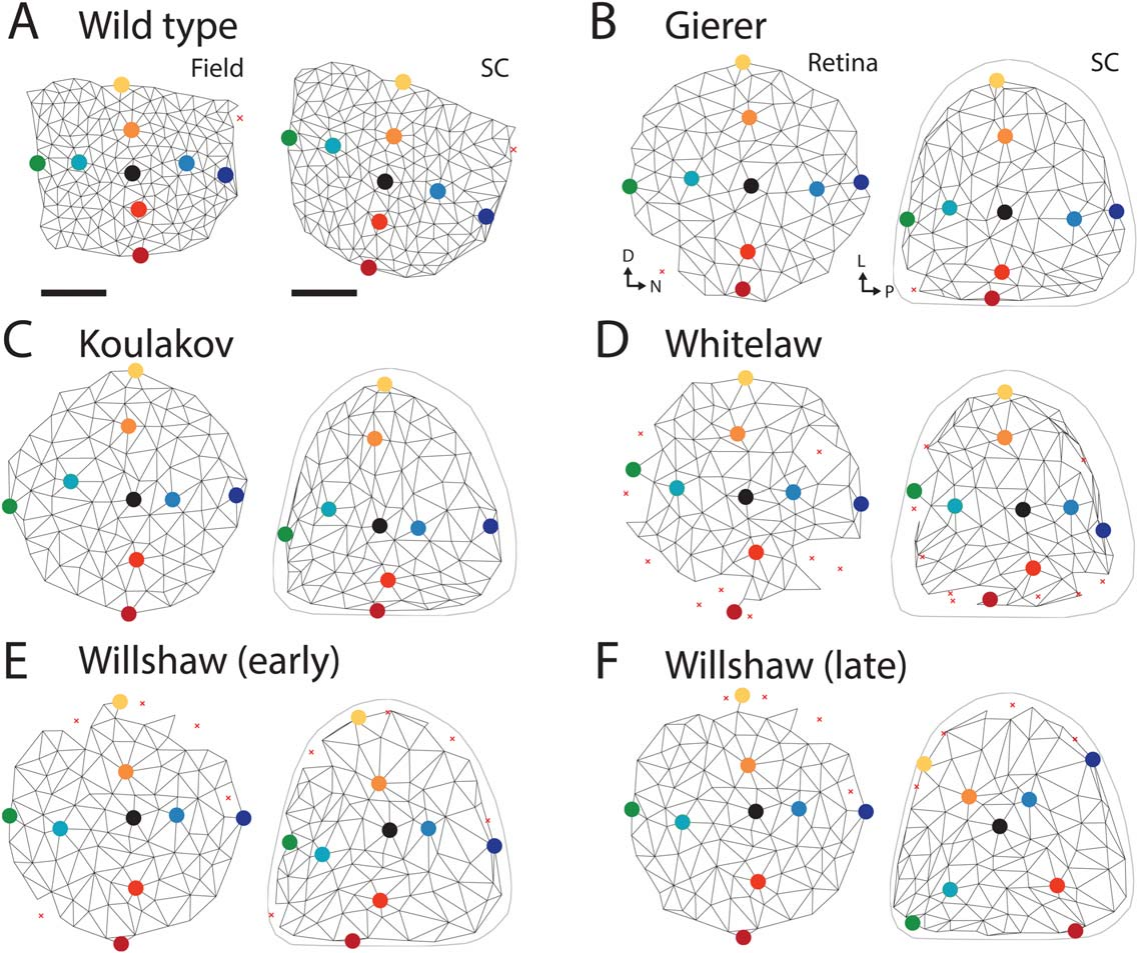
Lattice analysis of wild type map reveals a topographic projection. A lattice superposed over the retina (or the field) is deformed by the projection onto the SC. The projection of each node of the lattice is the averaged projections of nearby retinal neurons. Nodes are connected to their neighbors by black lines. Red crosses mark nodes in the Lattice analysis that were removed to maintain a locally ordered submap. The nine colored filled circles act as visual guides. (A) The adult wild type map acquired by intrinsic imaging shows a topographical map from field to the SC. The axes for the experimental data are flipped relative to simulated data, since nasal field projects on temporal retina. (B–E) Illustrative examples of the four main models are shown. (F) Extended Willshaw simulation (1,200,000 steps instead of 48,000), showing a rotation of the map.

**Figure 4:**
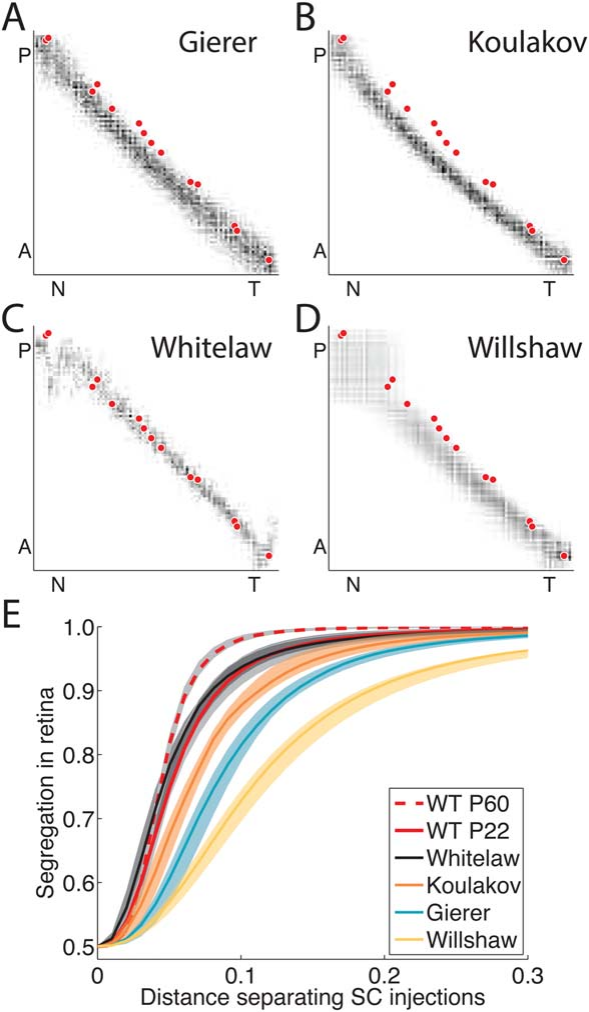
Topography and precision of the wild type map. (A–D) Projection from nasotemporal (NT) axis in the retina to the AP axis in the SC. The black 2D histogram shows modelled connections; red overlaid dots are experimental data (Brown et al., 2000). Only projections from the central third of the mediolateral axis of the retina are included. All models create a topographic map, but Gierer (A) and Koulakov (B) have a slight preference for anterior connections compared to the experimental map. Whitelaw (C) creates the most precise map, and Willshaw (D) the least precise map. (E) Retinal segregation of two retrograde injections (red and green) in the SC as a function of distance. The segregation measure is defined as the fraction of neurons whose closest neighbor has the same labelling; means no segregation of the two injections, 1 means complete retinal segregation (see Methods section). Red lines represent experimental data at P22 (solid) and adult P60 (dashed). Light gray regions indicate confidence intervals of experimental data; ranges of simulations are shown in transparent colors.

The connections from retina to SC in adult wild type mice form a topographic map as demonstrated by both electrophysiology and intrinsic imaging (Dräger and Hubel, 1976; Cang et al., 2008). By applying the Lattice method (Willshaw et al., 2014) to this data, which involved placing a grid over the retina (or field) and studying the deformation of its projection onto the colliculus, we could quantify global topographical order (Willshaw et al., 2014). The adult wild type mouse has a topographic map in which the largest ordered submap includes the entire field as shown in Figure 3. The global order was reproduced by all models, but the Whitelaw and Willshaw models had map defects due to edge effects (Table 4 and Fig. 4).

**Table 4:** Summary of Lattice Measure for the Largest Locally Ordered Submap

	Largest Ordered Submap Size	
Genotype / Model	Nodes (%)	Edges (%)
Wild type		
Experiment	98.3 *±* 2.1	99.5 *±* 2.1
Gierer	97.8 *±* 3.9	99.3 *±* 1.2
Koulakov	99.2 *±* 2.5	99.9 *±* 0.5
Whitelaw	59.8 *±* 8.4	88.1 *±* 2.9
Willshaw	79.6 *±* 9.1	94.8 *±* 2.3
*Isl2‐EphA3* ^ki/ki^		
Experiment	–	–
Gierer	99.0 *±* 2.2	99.7 *±* 0.7
	60.6 *±* 10.3	87.8 *±* 3.6
Koulakov	97.6 *±* 3.1	99.3 *±* 1.0
	51.9 *±* 11.8	81.0 *±* 7.0
Whitelaw	60.1 *±* 6.8	88.5 *±* 3.0
	29.0 *±* 7.8	73.8 *±* 4.2
Willshaw	84.8 *±* 7.5	95.8 *±* 2.6
	77.5 *±* 9.7	93.5 *±* 3.3
*Isl2‐EphA3* ^ki/+^		
Experiment	–	–
Gierer	94.7 *±* 7.0	98.3 *±* 2.1
	77.6 *±* 8.6	93.6 *±* 2.5
Koulakov	95.7 *±* 3.3	98.8 *±* 0.8
	79.6 *±* 11.9	93.1 *±* 5.2
Whitelaw	54.5 *±* 5.1	87.8 *±* 2.7
	42.9 *±* 7.7	81.5 *±* 3.8
Willshaw	86.9 *±* 7.0	83.0 *±* 5.6
	96.7 *±* 1.8	95.9 *±* 1.6
TKO		
Experiment	20.6 *±* 12.4	64.9 *±* 13.7
Gierer	0.4 *±* 0.7	20.0 *±* 5.4
Koulakov	6.9 *±* 7.5	38.9 *±* 12.8
Whitelaw	0.1 *±* 0.3	18.9 *±* 3.2
Willshaw	25.9 *±* 21.2	63.3 *±* 14.7
*Math5* ^−/−^		
Experiment	–	–
Gierer	27.9 *±* 8.4	73.6 *±* 4.2
Koulakov	77.2 *±* 8.8	93.3 *±* 3.1
Whitelaw	36.9 *±* 15.0	76.5 *±* 7.3
Willshaw	71.1 *±* 11.9	91.0 *±* 4.5

The size is given both as the percentage of edges in the largest ordered submap, and as percentage of nodes in the largest ordered submap that have retained all their edges compared to the full map. Values are given as mean *±* SD (*N* = 10). Where experimental intrinsic imaging data is available (Cang et al., 2008), the corresponding Lattice analysis values are reported (Willshaw et al., 2014). For *Isl2‐EphA3* ^ki/ki^ and *Isl2‐EphA3* ^ki/+^, the upper values are for the Isl2^−^ map, and the lower values the Isl2^+^ map.

We assumed that gradients were aligned with the standardized axis along which gradients are normally measured (NT, dorsoventral, AP, mediolateral). However, the experimental gradients may not align with these axes, as the visual field in the SC is rotated by about 19° (Dräger and Hubel, 1976; Willshaw et al., 2014). The simulated maps aligned with the axes, except for the Willshaw model which initially produced a map in the correct orientation [Fig. 3(E)], but then drifted gradually over time [Fig. 3(F)]. This drift occurred because both the ephrin‐A and ephrin‐B gradients in the SC were modifiable, and therefore, not locked to the AP and mediolateral axes as in the other models. The orientation stabilized so that the gradients were oriented diagonally across the SC, thus maximizing their length. The duration of the rotation was much longer (20 times) than the period of initial organization, so it is questionable whether this drifting orientation is relevant. However, we have no direct way of mapping simulation time onto real developmental time.

To assess the precision of order in the retinotopic map, Upton et al. (2007) developed a method by which dye is focally injected into the SC, and then retrogradely transported to the retina. Small focal labels in the retina indicate a precise mapping. The percentage of labelled retinal area is measured using contour analysis (Lyngholm et al., 2013; Sterratt et al., 2013). In wild type mice, the percentage of labelled retina decreased during development, indicating ongoing refinement of the map (Lyngholm et al., 2013), see also Table 5. We performed virtual retrograde injections to assess precision in the simulated maps. We found that the maps from the Koulakov, Whitelaw, and Gierer models had similar precision to P12 mice (Table 5). The Whitelaw model, however, showed large variations in retinal coverage due to map imperfections. The Willshaw model projections were more diffuse and closer to observations in P8 mice. Increasing the number of neurons in the simulations increased the precision of the map. However, with around 50,000 RGCs (Jeon et al., 1998; Salinas‐Navarro et al., 2009), and 12–22 subtypes (Sun et al., 2002; Kong et al., 2005; Völgyi et al., 2009; Sümbül et al., 2014), there might be around 2,300–4,200 of each RGC type, each potentially with their own map, which would be comparable to the 2,000 RGCs simulated here.

**Table 5:** Contour Analysis of Retinal Labelling from Retrograde Injections in the SC in Wild Type

	Retinal Coverage (%)
Experiment (P8)	11.1 *±* 9.1
Experiment (P12)	3.2 *±* 2.1
Experiment (P22)	2.6 *±* 1.1
Gierer	6.9 *±* 2.0
Koulakov	4.0 *±* 1.0
Whitelaw	6.1 *±* 8.8
Willshaw	13.0 *±* 1.2

Mean *±* SD of retinal coverage for 95% of the labelling is reported. Experimental data from Lyngholm et al. (2013).

To further characterize map precision, paired dye injections were made into SC to see how the retrogradely transported labels separated in the retina (Upton et al., 2007; Lyngholm et al., 2013). We performed equivalent virtual experiments: in Figure 4(E), the degree of segregation between the two retinal regions was plotted as a function of the separation of the “virtual” injections in the SC. The models were designed to represent development up until eye opening at P13 in mouse (McLaughlin et al., 2003), and no model reached the precision observed in P60/adult wild type mice. The Whitelaw model was the most precise and lay within the experimental range of what was seen at P22, followed by Koulakov, then Gierer, and the Willshaw model.

The difference in map precision can also be seen in the projection on the NT axis onto the AP axis [Fig. 4(A–D)]. Here, the Willshaw model has a wider diagonal (more spread out projections) while the Whitelaw model has the narrowest [Fig. 4(E)]. The Gierer and Koulakov models deviate from the diagonal, slightly favoring anterior connections. This was presumably due to the single repulsive gradient which, in combination with a weaker competition, makes posterior connections less favorable.

### Knock‐in of *EphA3*

About 40% of RGCs express *Isl2* in a “salt and pepper” fashion across the retina. *Isl2* represses an ipsilateral pathfinding program involved in deciding the laterality of RGCs in the ventral‐temporal crescent (Pak et al., 2004). *EphA3* is not endogenously present in retina, but by selectively knocking in *EphA3* in *Isl2*‐expressing RGCs, neighboring RGCs had largely different levels of EphA (Brown et al., 2000; Reber et al., 2004). Isl2^+^ RGCs had a higher *EphA* expression than their Isl2^−^ neighbors, and project more anteriorly into SC, where there was less ephrin‐A. Furthermore, the amount of knocked‐in *EphA3* could be doubled in a homozygous knock‐in compared to a heterozygous knock‐in.

**Figure 5:**
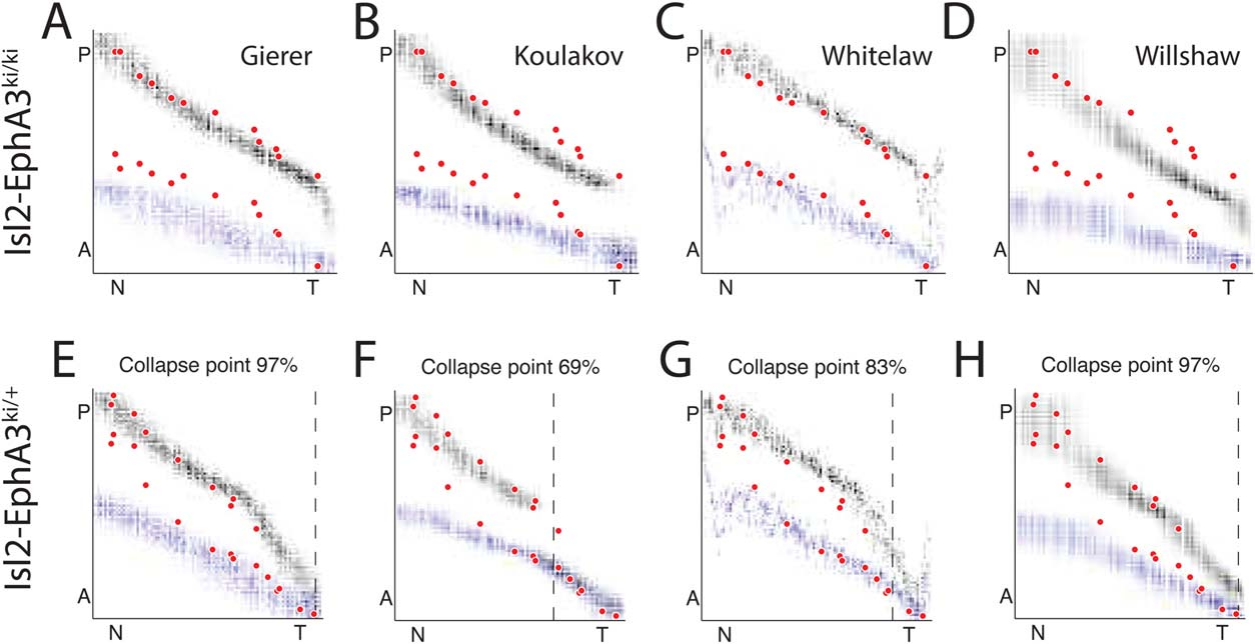
Map duplication when *EphA3* is added into the retina. In the homozygous *Isl2‐EphA3* knock‐in, the entire map is duplicated (top row). In the heterozygous knock‐in (bottom row), the nasal part of the map is duplicated, but appears to collapse at around 76% of the map. Red dots superimposed show experimental data taken from Figure 5 of Brown et al. (2000). Black shows connections from Isl2^−^ RGCs, blue shows Isl2^+^ RGCs with extra *EphA3*. Only projections from the central third of the mediolateral axis of the modelled retina are included. The Koulakov model shows a collapse point for the heterozygous knock‐in, the other models have a gradual merging of the two maps. No model has correct separation between the Isl2^+^ and Isl2^−^ maps in the SC for nasal projections.

These mutants were instructive in rejecting models based solely on Type I gradient mechanisms. See Choice of Models subsection in Methods for a description of Types I and II mechanisms. In mice with homozygous knock‐in of *EphA3*, the map from the retina split into two submaps (Fig. 5, red dots represent experimental data). A single anterograde injection along the NT axis in the retina generated two termination zones along the AP axis in the SC (Brown et al., 2000). However, the two maps had some overlap in the SC. A single retrograde injection into the anterior or posterior part of SC yielded one retinal termination zone, while an injection in the central part of the SC gave two termination zones in the retina. Below we discuss separately the homozygous and heterozygous knock‐in of *EphA3*.

#### Homozygous knock‐in of EphA3

All four models generated a double map for the *Isl2‐EphA3* ^ki/ki^ mutant; there were, however, subtle differences between the model results and experimental data. The Gierer, Koulakov, and Willshaw models placed the Isl2^+^ map (blue) anterior of the experimental data [red dots, Fig. 5(A,B,D)], and the Isl2^−^ submap appeared to dip down anteriorly at the temporal end. The Whitelaw model was optimized for the *Isl2‐EphA3* ^ki/ki^ phenotype and showed a good fit to experimental data over the majority of the NT axis [Fig. 5(C)]; the exception was for extreme temporal injections which, as in the other simulations, terminated more anteriorly.

**Figure 6:**
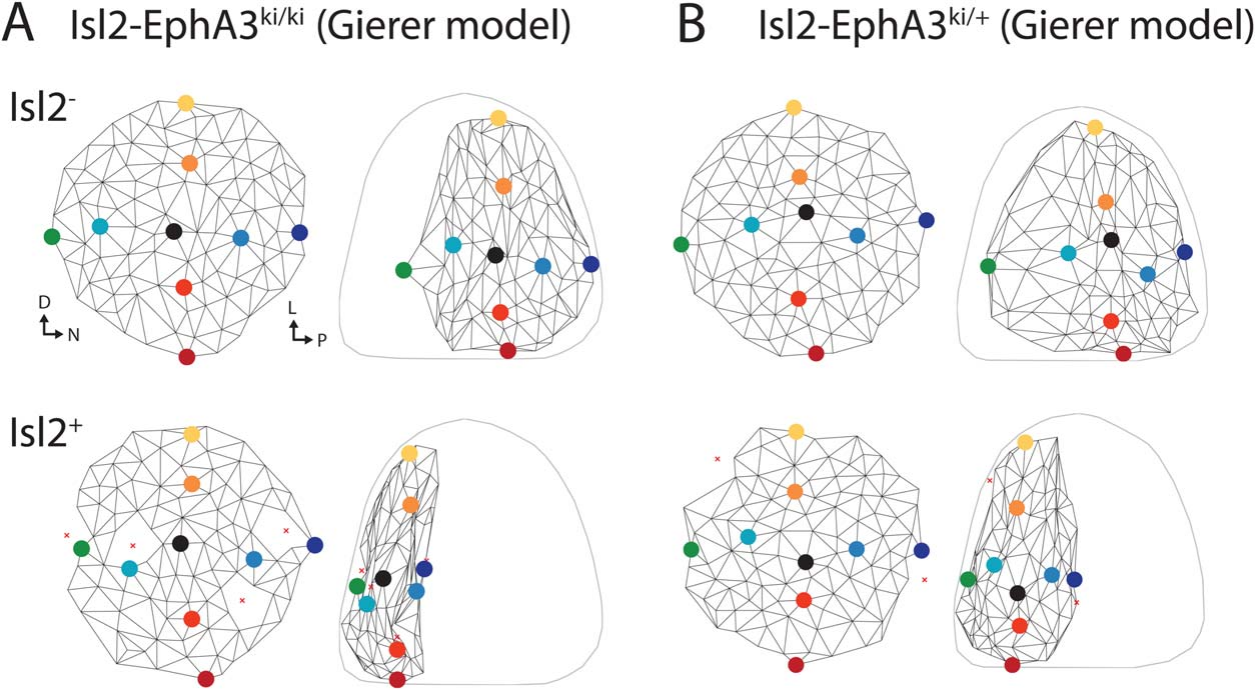
Lattice analysis of *Isl2‐EphA3* ^ki/ki^ and *Isl2‐EphA3* ^ki/+^. (A) The extent of the Isl2^+^ and the Isl2^−^ submaps for the *Isl2‐EphA3* ^ki/ki^ phenotype are illustrated with the Lattice analysis. Results are shown for the Gierer model and are representative of other three models. (B) In the *Isl2‐EphA3* ^ki/+^, the Gierer Isl2^−^ map shows expansion anteriorly, and compression posteriorly. The Isl2^+^ map is restricted to the anterior end, overlapping with the Isl2^−^ map, the Whitelaw, and Willshaw models look similar, the Koulakov has a slightly lower lattice density in the middle of the Isl2^−^ due to the collapse point (data not shown). See legend of Figure 3 for explanation of lattice plots.

The Lattice analysis showed that the Isl2^+^ and Isl2^−^ submaps were almost separated for all four models [Fig. 6(A)]. In the Koulakov model, this had the consequence that a single anterograde injection gave two termination zones, but a retrograde injection gave only a single termination zone. The lattices showed less order in the Isl2^+^ submap for the Whitelaw model than for the other models (Table 4).

**Figure 7:**
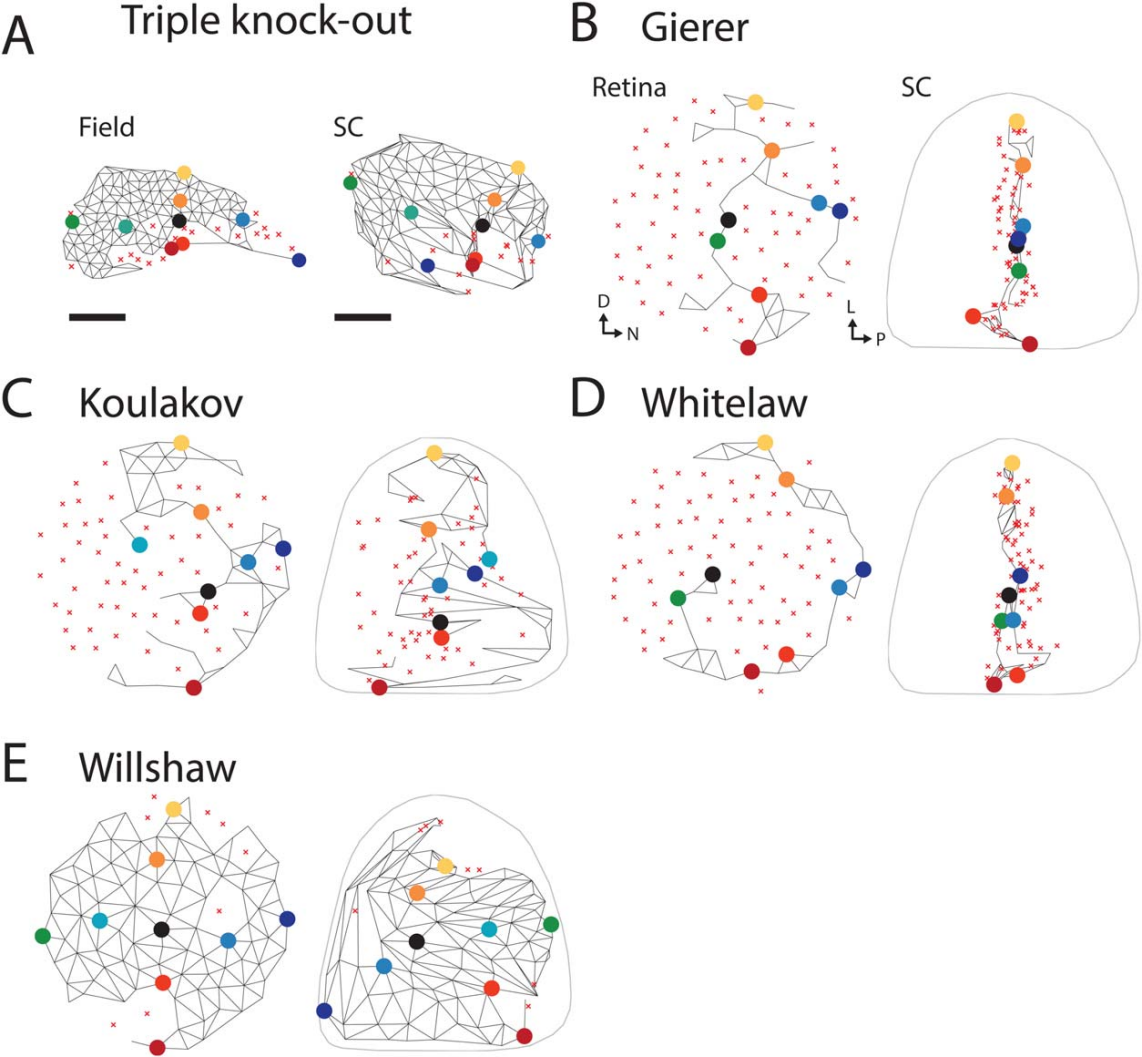
Lattice analysis of maps from TKO simulations reveals lack of global order. (A) Lattice analysis on intrinsic imaging data reveals order along the anteroposterior (AP) axis in the TKO (Willshaw et al., 2014). (B) The Gierer model lacks order along the AP axis. (C) The Koulakov model generates patches of local order, but no global order. (D) The Whitelaw model lacks order along the AP axis. (E) The Willshaw model produces large patches of order, but the map is in the incorrect orientation. See legend of Figure 3 for explanation of lattice plots.

#### Heterozygous knock‐in of EphA3

In the *Isl2‐EphA3* ^ki/+^ mutant, there was a double map in SC which collapses into a single map (Brown et al., 2000; Reber et al., 2004) in anterior SC: nasal retinal injections yielded two termination zones, while a temporal injection resulted in only one termination zone [Fig. 5(E), red dots]. The termination zones from the nasal anterograde injections were further apart in the *Isl2‐EphA3* ^ki/ki^ compared to the *Isl2‐EphA3* ^ki/+^. For retrograde injections, a single injection in the posterior SC generated two projection zones in the retina.

All four models could reproduce the anterograde tracing experiment in which a nasal injection yielded two termination zones in the SC, and a temporal injection gave only one termination zone. However, they deviated from experimental results in the details. For the nasal injection, the two resulting termination zones were further apart than in experiments. There was also a difference between how the maps merged in the models compared to the experiments. For the Gierer, Whitelaw, and Willshaw models, the two maps gradually merged [Fig. 5(E,G,H)], while the Koulakov model was the only one to exhibit a collapse point similar to what was seen in experiments [Fig. 5(F), 70 *±* 3% along NT axis]. The merge points for the three models were located at: Gierer 95 *±* 3% (7/10 simulations, 3 simulations did not merge); Whitelaw 84 *±* 2%; and for Willshaw 86 *±* 8% (9/10 simulations, 1 simulation did not merge). None of the models produced two projections zones in the retina for retrograde injections in posterior SC. In the Koulakov model, the collapse point was seen in the lattice, where the Isl2^−^ map was stretched (data not shown) in the center. For all models, the Isl2^+^ submap does not extend as far posteriorly as would be expected from experiments [Fig. 6(B), showing Gierer model]. The Lattice analysis looks very similar for Whitelaw and Willshaw (data not shown), with a stretching of the anterior part of the Isl2^−^ submap.

### TKO of *ephrin‐A*

**Figure 8:**
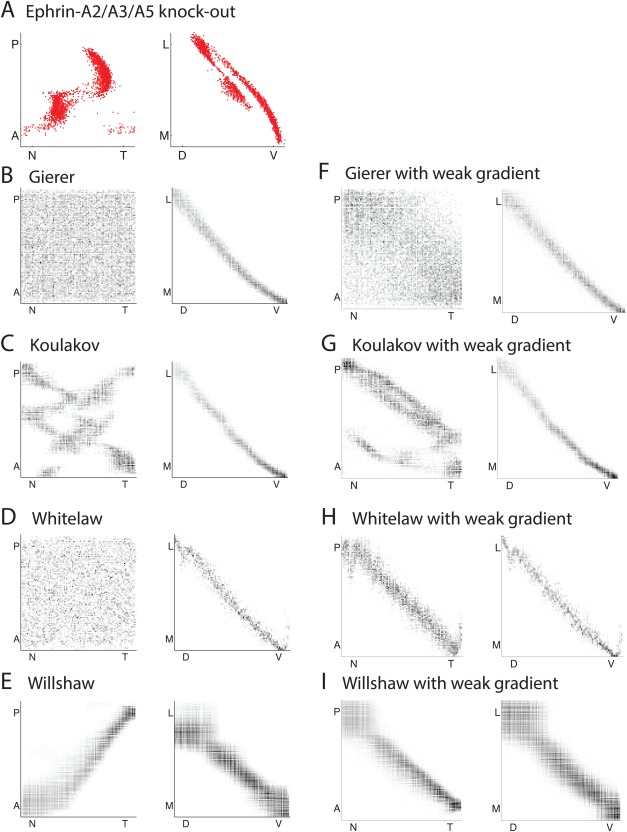
NT and dorsoventral projections in TKO mice. (A) Data from experimental intrinsic imaging (Cang et al., 2008) showing how the visual field projects onto the AP axis, here only the central third of the retina along the dorsoventral axis is used. Similarly for the visual field onto the mediolateral plot, where only the central third of the retina along the NT axis is used. (B) The Gierer model maintains order along the mediolateral axis, but shows no order along NT axis. (C) In the Koulakov model, correlated retinal activity joins the projections from neighboring RGC axons together, creating patches of local order. (D) The Whitelaw model cannot produce order along the AP axis. (E) The Willshaw model induces gradients in the SC, forming order along the AP axis, but also destroying part of the order along the mediolateral axis in the process. In the case shown, the AP polarity of the map is reversed. (F) The Gierer model with a weak AP gradient (*K* = 0.01) only has a slight increase in the density of projections on the diagonal. The Koulakov maps with a weak gradient show more order, and large variations between runs. (H) The Whitelaw model with a weak gradient shows a complete diagonal. (I) The Willshaw model only needs the weak gradient to establish polarity and form a complete diagonal.

By knocking out *ephrin‐A2*, *ephrin‐A3* and *ephrin‐A5* (TKO) in mouse, all ephrin‐A ligands, which provide information about NT mapping, were absent. The resulting map retained mediolateral order, but initial analysis suggested very little order in the AP direction, with patches that coactivated when one region of the retina was stimulated (Cang et al., 2008). A more detailed analysis of the topography using the Lattice method (Willshaw et al., 2014) revealed a map with more global order in the AP direction than initially reported [Fig. 7(A)]. In these TKO maps, 10% of the retinal positions projected to more than one circumscribed area of colliculus, suggesting the presence of ectopic projections (Pfeiffenberger et al., 2006). Figure 8(A) shows the intrinsic imaging data projected onto the AP and mediolateral axes, where the ectopic projections and map distortions are visible.

The TKO maps from both the Gierer and Whitelaw models showed no order along the AP axis [Fig. 8(B,D)]. The Lattice analysis looked similar for the two models [Fig. 7(A,C)], with no regions that retained their order when projected to the SC; instead the grid points were all centered along the AP axis. This was also reflected in the relatively small size of the largest ordered submaps (Table 4). The lack of order in the Gierer model was consistent with the lack of interactions between presynaptic axons other than competition. In the Whitelaw model, an ordered map might have been expected, since a mechanism of axon–axon interactions, possibly mediated by neural activity, can produce ordered maps, but on its own cannot specify global orientation (Willshaw and von der Malsburg, 1976). The lack of order suggests that the specific activity mechanism implemented in the model was weak. This was consistent with the model's performance on the *Isl2‐EphA3* ^ki/+^ mutant, where there was no collapse point.

In addition to competition, the Koulakov model also has a neural activity term that allowed for interaction between presynaptic axons, albeit indirectly through their postsynaptic targets. The resulting map showed patches of local order, where neural activity had joined projections of neighboring neurons [Fig. 8(C)]. Some regions of the NT axis projected onto two or more distinct regions of the AP axis, which was a hallmark of ectopic projections. The Lattice analysis detected ordered patches (Table 4), and linked them together to display the largest locally ordered submap [Fig. 7(C)], but it was much smaller than experimental submaps. There was no global order along the AP axis in the Koulakov model maps, and the polarity of the largest ordered submap varied between different runs.

Despite lacking global polarity cues, the Willshaw model could induce considerable order into the largest locally ordered submap [Fig. 7(E)], matching that seen in experiments (Table 4). In addition to disrupted AP order, the Willshaw model occasionally failed to reproduce correct mediolateral order [Fig. 8(E)], which was not the case for the other models. Since collicular gradients adapted during simulations in the Willshaw model, some of the order was lost in the dorsoventral axis as the EphB and ephrin‐B gradients were modified when the system tried to induce ephrin‐A gradients into an SC that initially lacks ephrin‐A.

One possible explanation for the residual global AP order in the TKO animals is that there are gradients of molecules other than EphAs and ephrin‐As along the retinal NT and collicular AP axes which provide weak guidance information to axons. In mouse, Neuropilin 2 and Semaphorin 3F are expressed in increasing NT and AP gradients in the retina and SC, respectively (Claudepierre et al., 2008). Collapse assays showed that temporal RGC axons collapsed more frequently than nasal axons in the presence of Semaphorin 3F (Claudepierre et al., 2008), though the fraction of axons collapsing was low (4% vs. 12%).

If this hypothesis is true, a fairer test of the models is to introduce a weak gradient over the rostrocaudal axis of the SC in the homozygous TKO simulations. A simple way of simulating a weak interaction between retina and colliculus is to replace the wild type collicular ephrin‐A gradient with a molecule having the same profile, scaled by a factor *K <* 1.

**Figure 9:**
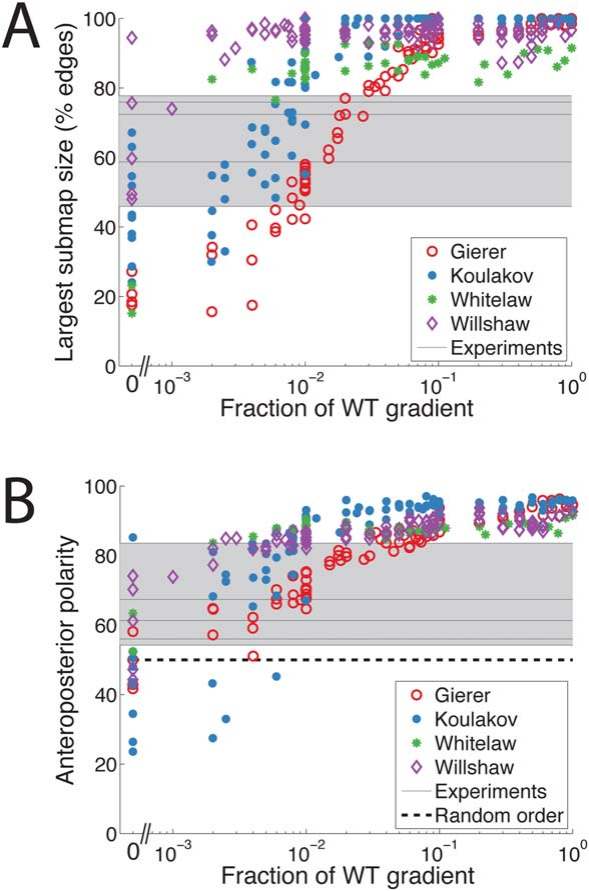
Recovering AP order in the output maps of the models by reintroducing a weak ephrin‐A gradient into TKO. (A) Percentage of edges in the largest ordered submap as a function of ephrin‐A reintroduced. (B) AP order as a function of ephrin‐A reintroduced. Dashed black line shows AP order for random maps. The gray region defines the range of experimental values observed. Black lines indicate individual experiments.

Figure 9 shows how the local order and the order along the AP axis vary as the strength of the weak gradient *K* is scaled down from 1, the wild type value. Between *K* = 1 and *K* = 0.1, both measures remained broadly unchanged for all models.

Between *K* = 0.1 and *K* = 0.01, all models except for Gierer showed better quality maps than in the homozygous TKO maps. Between *K* = 0.01 and *K* = 0.002, the quality of the results from Koulakov was in the range of the homozygous TKOs and those from Gierer were worse; the other two models still display higher quality maps.

The figures show that the spread of local and global order of the homozygous TKO maps is represented by a value of *K* ranging from 0.03 to 0.008 for the Gierer model and 0.01 to 0.002 for the Koulakov model. It is difficult to know how weak this gradient is relative to wild type because lack of information about gradients and effective interaction strengths prevents us from knowing whether a value of *K* = 1 corresponds to the wild type.

### *Math5* ^−/−^ Knock‐Out

RGC axons growing into the SC appear to compete with each other for postsynaptic targets (Gosse et al., 2008). One way to investigate the effect of axonal competition on map formation is to reduce the number of innervating RGCs. The *Math5* ^−/−^ mutant has 5–10% of the number of wild type RGCs (Triplett et al., 2011), and thus, the remaining RGCs experience reduced competition from their neighbors. The mapping in the context of reduced competition was disrupted: instead of innervating the entire SC, the projections were focused in the anteromedial region (Triplett et al., 2011). It is still an open question whether the map which forms is topographic.

**Figure 10:**
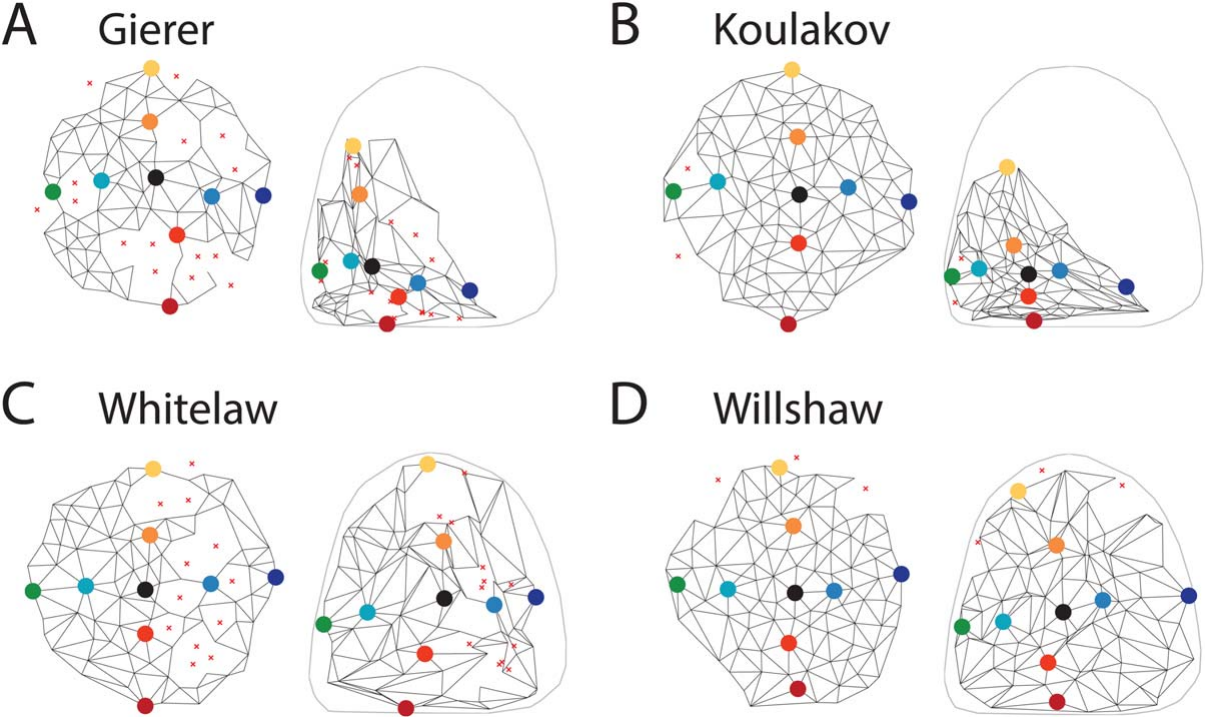
Lattice analysis of *Math5* ^−/−^ simulations. The Gierer (A) and Koulakov (B) models show a anteromedial localization of the maps in the SC for *Math5* ^−/−^, with the Koulakov map being more ordered (78.1 *±* 8.4 vs 28.4 *±* 7.6 nodes in largest ordered submap). Both the Whitelaw (C) and Willshaw (D) models fail to produce the *Math5* ^−/−^ phenotype, instead projecting across the entire SC.

The Gierer model captured the anteromedial confinement of the projection [Fig. 10(A)]. All RGC axons had highest affinity anteriorly, and it was only through competition that some of them were pushed more posteriorly. However, with the reduced population in the model, the remaining neurons could terminate more anteriorly than they would normally do. The Koulakov model also reproduced the anteromedial bias of the projection [Fig. 10(B)]. Comparing the Gierer and Koulakov maps, we saw that they covered a similar fraction of the SC (99% of synapses cover 48.5 *±* 0.4 vs. 50.0 ± 0.4%). There was, however, more order in the Koulakov map than in the Gierer map (Table 4). The RGCs in the Whitelaw model innervated the entire SC [Fig. 10(C)] because postsynaptic normalization ensured that all SC neurons receive input. There was some order retained in the largest ordered submap, but less so than in the Koulakov model. Like the Whitelaw model, the Willshaw model also contained mechanisms that ensured that the entire SC was innervated [coverage 98.5 *±* 0.4%, Fig. 10(D)], and most of it was topographically ordered (Table 4).

### Summary

In this study, the Gierer, Koulakov, Whitelaw, and Willshaw models of retinotopic map formation have been evaluated quantitatively on a set of phenotypes. In each model, the same set of parameter values was used for all simulations. Three of the four models were fitted to one of the phenotypes: Gierer *Math5* ^−/−^, Koulakov *Isl2‐EphA3* ^ki/+^, and Whitelaw *Isl2‐EphA3* ^ki/ki^. The Willshaw model did not require any additional fitting.

Our results are summarized in Table 6. We find that all models can account for wild type maps and the homozygous *EphA3* knock‐in maps. The Koulakov model was the only model to generate a collapse point in *Isl2‐EphA3* ^ki/+^ maps. The Willshaw model was the only model to produce the internal order seen in TKO maps without any extra cues, but it does not capture the global polarity. By adding a weak gradient (which might correspond to retinal Neuropilin 2 and collicular Semaphorin 3F) all models could produce internal order and global polarity. The Gierer and Koulakov models can produce the compression of the map into the anteromedial part of SC seen in the *Math5* ^−/−^ phenotype.

**Table 6:** Summary of Model Evaluation

Genotype	Gierer	Koulakov	Whitelaw	Willshaw
Wild type	✓	✓	✓	* ✓
*Isl2‐EphA3* ^ki/ki^	Isl2^+^ misfit	Isl2^+^ misfit	* ✓	Isl2^+^ misfit
*Isl2‐EphA3* ^ki/+^	No collapse, Isl2^+^ misfit	* Isl2^+^ misfit	No collapse, Isl2^+^ misfit	No collapse, Isl2^+^ misfit
TKO (no gradient)	No patches	Patches but no global order	No patches	Global order but no polarity
TKO (weak gradient)	No patches	✓	No patches	Ordered map
*Math5* ^−/−^	* ✓	✓	Normal map	Normal map

Asterisk (*) denotes which phenotype the model was optimized for.

## DISCUSSION

Since the initial experiments by Sperry (1943, 1963), the retinocollicular or retinotectal projection has been used as a model system for the development of ordered nerve connections, many computational models have been proposed. Several reviews have synthesized properties of computational models proposed in the last 40 years to account for the development of retinotopic maps (Swindale, 1996; Goodhill and Xu, 2005; Goodhill, 2007). However, it has been difficult to assess and compare models, because either different models were formalized in incompatible ways or they were designed for a specific set of data or key experimental data was not available to test them.

Therefore, before embarking on generating new models, we aimed to explore rigorously whether any of the four models chosen could account for all known data on retinocollicular maps in mouse. To do this, we have developed an open computational framework to compare, quantitatively, the results from theoretical models of retinotopic map formation against experimental data. We chose a set of well‐documented experimental data for the mouse visuocollicular system as reference experimental data. Exhaustive testing of all previous retinotopic map formation models is infeasible and so we selected four representative models that we believe collectively sample the major mechanisms hypothesized for map formation. In choosing models, we had to eliminate those which were not described in sufficient detail to enable us to simulate a 2D version and those in which there was no explicit representation of gradients. The four models chosen are: the Gierer model (1983), the Koulakov model (2006–2011), the Whitelaw model (1981), and the Willshaw model (1977–2006). The previously published 1D versions of both the Gierer and the Whitelaw models required considerable extension to enable them to reproduce 2D maps.

### Summary of Model Performance

1. All models could replicate wild type maps and produce qualitatively correct double maps seen in *Isl2‐EphA3* ^ki/ki^ mice. The Whitelaw model produced the best match to the *Isl2‐EphA3* ^ki/ki^ maps, although its parameters were optimized for this condition.
2. The Koulakov model alone reproduced a collapse point in *Isl2‐EphA3* ^ki/+^ mice, due to the strong activity‐dependent mechanism. The relative contribution of activity‐dependent mechanisms in the Whitelaw model was too weak to generate collapse points. Both the Gierer and Willshaw models lack a mechanism that conveys information about distance between pairs of retinal cells independently of gradients and so were not expected to reproduce the collapse point.
3. No model could account for the consistent, residual global order along the rostrocaudal axis in maps when all ephrin‐A ligands were removed (Cang et al., 2008; Willshaw et al., 2014). However, both the Koulakov and Willshaw models produced some order along the anterior–posterior axis, though its origin was quite different in the two cases: from correlated neuronal activity (Koulakov); from the spatial continuity enforced by diffusion of collicular markers (Willshaw). By reintroducing a weak rostrocaudal gradient back into the SC, a largest ordered submap consistent with experiments can be produced by the other two models.
4. The Gierer and Koulakov models reproduce the *Math5* ^−/−^ phenotype where the projection is restricted to one portion of the colliculus. In the Whitelaw model, the strong postsynaptic normalization counteracted the effect of the Type II mechanism to cluster axons at the temporal end; in the Willshaw model, diffusion of collicular labels caused the projection to spread, across the entire colliculus.

### Insights into Mechanisms of Mapping

We now summarize what we have learnt about the mechanisms of map formation and what components any new model should possess. We do this in terms of the five component mechanisms mentioned in the Introduction section.

### Chemoaffinity

We found that two combinations of chemoaffinity account for the formation of wild type maps and the *Isl2‐EphA3* ^ki/ki^ maps.

- Type II affinity (Prestige and Willshaw, 1975) with single set of gradients and a competitive mechanism (Gierer, Whitelaw, and Koulakov); Gierer and Koulakov also gives a restricted projection in the *Math5* ^−/−^ case.
- Type I affinity with a single set of retinal gradients together with variable collicular gradients (Willshaw)
- Models using countergradients cannot be ruled out but those using fixed gradients with no plasticity are excluded by the *Isl2‐EphA3* ^ki/ki^ data.

#### Spontaneous Neural Activity and Hebbian Synapse Formation

The main effect we observed in introducing a mechanism involving neural activity is that it enables the Koulakov model to reproduce the collapse point in the *Isl2‐EphA3* ^ki/ki^ map. Activity seems also to be necessary for the refinement of initial axonal arbors (Lyngholm et al., 2013). The representation of neural activity in both the Whitelaw and Koulakov models is quite abstract and so is hard to relate to experimental data. A more explicit representation (e.g., spike times or bursting activity of neurons) would allow retinal wave data to be used more directly in models and allow for a more direct comparison with *β2* ^−/−^ mice and other activity‐altering genotypes.

#### Competition

All models tested incorporate a competition mechanism to give flexibility in the map. In the context of the neuromuscular system, competition mechanisms have been classified as consumptive competition (for neurotrophic factors) or interference competition, either for space, or where axons have direct negative interactions (van Ooyen, 2001). By manipulating expression levels of the neurotrophin BDNF in individual cortical neurons, it has been shown that BDNF helps the cells compete for inputs, and thus acts as a target for consumptive competition (English et al., 2012). Since BDNF and TrkB are expressed in the colliculus and retina respectively (Marler et al., 2008), there is, therefore, consumptive competition in the retinocollicular mapping. Theoretical competition rules which maintain the total synaptic weight assigned to all the synapses of a neuron at a constant level can be seen as an approximate implementation of consumptive competition; of the models studied the Whitelaw and Willshaw have this mechanism. The Koulakov model has a stochastic implementation of the mechanism. In contrast, the Gierer model has a form of competition more akin to direct negative interactions, for which we are not aware of any direct experimental evidence in the retinocollicular system. Manipulating competition rules in models could be used to check the intuition that reducing the expression in a portion of the SC might be expected to magnify the map from the retina in this region.

#### Ordering of Fibers in the Optic Tract

None of the models examined incorporates such a mechanism although in the original version of the Willshaw model (von der Malsburg and Willshaw, 1977), it was proposed that the fiber ordering could specify the overall orientation of the map. Evidence for ordering across the mediolateral dimension of the tract (Plas et al., 2005) could be used in future models. These would have to incorporate the three dimensions of fiber growth and innervation which so far have been neglected in models.

#### Axon–Axon Interaction

Here, we mean chemospecific signalling between RGC axons in the colliculus, either directly, as modelled by Yates et al. (2004) and Gebhardt et al. (2012), or indirectly in the Willshaw model through the labels induced from retinal axons into the colliculus. In direct interactions, Eph receptors on growing axons are activated by ephrin ligands on nearby retinal axons and the strength of this effect is supposed to grow as more axons fill the colliculus. Given a choice, temporal axons prefer growing on temporal retinal substrate, while nasal axons grow on both temporal and nasal retinal substrate (Bonhoeffer and Huf, 1985) and there is also direct evidence for axon–axon interactions from time lapse imaging of interactions between growing RGCs (Raper and Grunewald, 1990) as well as modelling arguments (Weth et al., 2014). Gebhardt et al. (2012) included direct axon–axon interactions in a model with gradients of retinal Eph and collicular ephrin and countergradients of retinal ephrin and collicular Eph. Without axon–axon interactions, the parameters of the gradients and countergradients had to be matched to produce wild type maps. Axon–axon interactions could compensate for this, although this may depend on a precise matching of parameters (Sterratt, 2013). Nevertheless, this demonstrates that axon–axon interactions may confer flexibility on map formation, even without competition. As we did not include countergradients, direct chemospecific axon–axon interactions were beyond the scope of our study, though they can be modelled using our pipeline.

The indirect axon–axon interactions in the Willshaw model, coupled with competition and a Type I affinity mechanism, gave very robust map formation—more robust to knockout of *Math5* ^−/−^ and *ephrin‐A*s than the experimental phenotypes. In the case of *Math5* ^−/−^, this robustness appears to be due to the Type I affinity mechanism. Once the collicular gradients have been set up, there is no part of the colliculus which is preferred by all axons. In contrast, in models with competition and Type II affinity, all axons prefer anterior colliculus; in the *Math5* ^−/−^ knockout, competition is not strong enough to then force out the less‐repelled nasal axons, as in wild types.

In summary, each model we examined had a mechanism of chemoaffinity and competition, and two models also had a mechanism representing neural activity and synaptic plasticity. The models accounted for most of the experimental data we examined using, within each model, the same parameter values for all of the genotypes. The main class of result that was not accounted for was the residual order seen in the homozygous TKO map, although these data could be fitted by an additional weak gradient. This could be provided, for example, by retinal and collicular gradients of Neuropilin 2 and Semaphorin 3F in mouse (Claudepierre et al., 2008), or possibly by repulsive guidance molecule, which in chick is expressed in a graded fashion and repels temporal RGC axons (Monnier et al., 2002). Another candidate is engrailed which is expressed in an AP gradient in chick tectum (Wizenmann et al., 2009; Stettler et al., 2012). This does not exclude the possibility that other factors, such as time of axon arrival, are involved in generating NT map polarity. We also need to consider that there could be other parameter sets for the four models tested, or the possibility of a model not included in the study, which would perform better. By restricting ourselves to optimizing for only one mutant phenotype, we have saved a set of “unseen” phenotypes to validate the model against. Another strategy would be to optimize for all experimental data simultaneously, but then there is no unused data to validate the models against.

### Experimental Considerations

As most experimental work in topographic map formation is now undertaken in mouse, we focused on curating the experimental data available in the literature including in wild type, *Isl2‐EphA3* ^ki/ki^, *Isl2‐EphA3* ^ki/+^, triple *ephrin‐A2,A3,A5* knock‐out, and *Math5* ^−/−^. In the *β*2^−/−^ mutant, activity has been disrupted by knocking‐out the *β*2 subunit of the nicotinic acetylcholine receptor (McLaughlin et al., 2003). This leads to larger termination zones of labelled axons in the SC, evident from around P4 (Lyngholm et al., 2013), and this effect would be interesting to investigate in future studies. We found that although there are many other documented disruptions to the retinotopic map, often there were few quantitative characterizations of the data, though this may partly be due to the limitations of experimental techniques and the variability of phenotypes. For example, a common phenotype observed in mutant mice is that of ectopic projections (Frisén et al., 1998; Feldheim et al., 2000). Here the raw data are images of colliculi stained by DiI transported by axons from retinal injection sites. Each individual has only one injection site and it would appear that there is considerable variability between individuals, so it is not possible to construct one composite map, as in the case of the knock‐in mutants. To move from a qualitative to a quantitative characterization of ectopic projections would require significant effort and, ideally, the availability of raw image data would allow for various methods of determining the location of dye spots to be tested.

The ability to obtain whole maps from individuals using functional imaging gets around the issues of interindividual variability, though it brings with it the problem of inferring anatomy from functional data. Ectopic projections defined functionally have been analyzed quantitatively in TKO Fourier imaging data (Willshaw et al., 2014); applying this technique to the ephrin‐A knockout data (Frisén et al., 1998; Feldheim et al., 2000) may prove fruitful.

Our modelling is dependent on (and limited by) quantitative characterization of the molecular gradients, notably retinal EphA receptors (Reber et al., 2004). Our best guesses of parameter values for the remaining Eph and ephrin gradients (Table 3) can be replaced with experimental findings once they become available. Currently, we have excluded countergradients from our models because (a) there is limited data about their expression levels, and (b) recent theoretical findings suggest that competition and countergradients can be traded off against each other (Sterratt, 2013).

To investigate the role of activity in the formation of a collapse point in *Isl2‐EphA3* ^ki/+^, it might be instructive to combine this mutant with *β2* ^−/−^ mice, where spontaneous activity is perturbed significantly (Stafford et al., 2009). It would be interesting to assess whether the two maps normally seen in *Isl2‐EphA3* ^ki/ki^ mice converge into one, or if the collapse point in the maps of *Isl2‐EphA3* ^ki/+^ mice moves. Unfortunately, *β2* ^−/−^ maps are inherently diffuse, so it might not be possible to separate the two cases in the combined mutants.

Finally, one limitation of our current approach is that although it provides full access to the developmental time course, currently we have limited developmental dynamics from the experimental system. We might expect that during the critical period of map formation in mouse, while the map is changing, other aspects of the system change too. For example, currently, we assume that molecular gradients are fixed, but these might flatten over time (Rashid et al., 2005). This could change the balance between mechanisms driven by activity and chemical cues.

### Future Work and Challenges

There are a number of directions in which the work can be taken:

1. While a combination of chemoaffinity, neural activity, and competition accounts for the data (within the limits stated), it may be that other combinations also comprising mechanisms of fiber preordering and/or axon–axon interaction can also account for the data. Then it should be possible to provide predictions to distinguish between the different possible models.
2. For each of the four models, we have found a set of parameter values that can be used to produce satisfactory maps on our current data sets. The challenge would then be to test out these models using the same set of parameter values on new data when available. It may also be worth exploring if there are other parameter values for the models that can perform similarly, or better.
3. Quantifying data from ephrin‐A knock‐outs and challenging the models with this data. The interindividual variability will prove a challenge; the question is how to match a distribution of models to a distribution of data.
4. Assessing map development throughout the developmental timeline. This requires data of both gradients and maps at different ages.
5. Mapping the mechanisms present in models onto lower level mechanisms. The models in the article are formulated at a fairly high level of abstraction (e.g., competition) and it would be desirable to investigate how these mechanisms might be implemented in more detail.

Unbiased quantitative evaluations of existing models using the framework that we have developed will allow us to see how the different models perform, and will help us guide future modelling efforts. Using a curated set of experimental data makes it easier to test a computational model and, when new experimental data becomes available, predictions can be generated on all models. We hope that our open‐access pipeline will inspire further unification of models to help comparison, and increase reproducibility (Stevens et al., 2013).

## Conflict of interest

The authors declare no competing financial interests.
